# Association of the Affordable Care Act Medicaid Expansion with Trauma Outcomes and Access to Rehabilitation among Young Adults: Findings Overall, by Race and Ethnicity, and Community Income Level

**DOI:** 10.1016/j.jamcollsurg.2021.08.694

**Published:** 2021-10-14

**Authors:** Gregory A Metzger, Lindsey Asti, John P Quinn, Deena J Chisolm, Henry Xiang, Katherine J Deans, Jennifer N Cooper

**Affiliations:** Centers for Surgical Outcomes Research (Metzger, Asti, Quinn, Chisolm, Deans, Cooper), Child Health Equity and Outcomes Research (Metzger, Asti, Quinn, Chisolm, Deans, Cooper), Pediatric Trauma Research (Xiang), and Injury Research and Policy (Xiang), Abigail Wexner Research Institute, Department of Pediatric Surgery (Deans), Nationwide Children’s Hospital, Departments of Surgery (Metzger, Deans) and Pediatrics (Chisolm, Xiang, Cooper), Medical Student Research Program (Quinn), College of Medicine, Divisions of Health Services Management and Policy (Chisolm), and Epidemiology (Cooper), College of Public Health, The Ohio State University, Columbus, OH.

## Abstract

**BACKGROUND:**

Low-income young adults disproportionately experience traumatic injury and poor trauma outcomes. This study aimed to evaluate the effects of the Affordable Care Act’s Medicaid expansion, in its first 4 years, on trauma care and outcomes in young adults, overall and by race, ethnicity, and ZIP code-level median income.

**STUDY DESIGN:**

Statewide hospital discharge data from 5 states that did and 5 states that did not implement Medicaid expansion were used to perform difference-in-difference (DD) analyses. Changes in insurance coverage and outcomes from before (2011–2013) to after (2014–2017) Medicaid expansion and open enrollment were examined in trauma patients aged 19 to 44 years.

**RESULTS:**

Medicaid expansion was associated with a decrease in the percentage of uninsured patients (DD −16.5 percentage points; 95% CI, −17.1 to −15.9 percentage points). This decrease was larger among Black patients but smaller among Hispanic patients than White patients. It was also larger among patients from lower-income ZIP codes (p < 0.05 for all). Medicaid expansion was associated with an increase in discharge to inpatient rehabilitation (DD 0.6 percentage points; 95% CI, 0.2 to 0.9 percentage points). This increase was larger among patients from the lowest-compared with highest-income ZIP codes (p < 0.05). Medicaid expansion was not associated with changes in in-hospital mortality or readmission or return ED visit rates overall, but was associated with decreased in-hospital mortality among Black patients (DD −0.4 percentage points; 95% CI, −0.8 to −0.1 percentage points).

**CONCLUSIONS:**

The Affordable Care Act Medicaid expansion, in its first 4 years, increased insurance coverage and access to rehabilitation among young adult trauma patients. It also reduced the socioeconomic disparity in inpatient rehabilitation access and the disparity in in-hospital mortality between Black and White patients.

For Americans aged 19 to 44 years, unintentional injuries and homicide were collectively responsible for more than 50% of deaths in 2010–2019, making young adults more at risk of dying from traumatic injury than from the next 3 leading causes of death combined.^1^ Before the Affordable Care Act (ACA), > 30% of adult trauma patients were uninsured at the time of injury, putting them at an increased risk of experiencing poor outcomes.^2^ Pre-ACA studies examining differences in outcomes after traumatic injury found that patients of low socioeconomic status, uninsured patients, and racial or ethnic minority patients were less likely to have access to post-discharge care and more likely to experience both in-hospital and long-term mortality.^3–7^ In addition, being uninsured was independently linked to shorter length of stay and increased likelihood of requiring readmission after discharge.^8,9^

The mechanisms driving disparities in access to care and outcomes after traumatic injury are complex, with potential contributors, including health insurance coverage, but also many other systemic, cultural, and geographic factors.^10^ The ACA was intended to improve access to healthcare and health outcomes by extending health insurance coverage to millions of previously uninsured Americans.^11^ In addition to a health insurance mandate and newly created subsidies, the ACA included a provision that required states to expand Medicaid coverage starting in January 2014 to all nondisabled adults aged 19 to 64 years with incomes up to 138% of the federal poverty level. However, a ruling by the Supreme Court in 2012 declared that the decision to expand Medicaid stood with the individual states.^12^ As of January 2021, 38 states had implemented ACA Medicaid expansion. The American College of Surgeons worked closely with legislators to ensure that the ACA would support efforts to improve the quality of trauma care across the US by increasing health insurance coverage in vulnerable populations, by stabilizing the trauma network through policies aimed at reducing uncompensated care, and by strengthening the healthcare system through an emphasis on research.^13^ Although implementation of the ACA has led to increases in insurance coverage across the country, the effect of the ACA on racial, ethnic, and socioeconomic disparities in insurance coverage has been uneven, partly due to the lack of Medicaid expansion in some states. In 2010, 46.5 million nonelderly adults across the US were uninsured, including one-third of Hispanic Americans and 20% of Black Americans. As of 2018, the uninsured rate had decreased to 10.4% among nonelderly adults, but Hispanic adults remained more than 2.5 times more likely, and Black adults remained 1.5 times more likely, than White adults to be uninsured.^14^

The impact of trauma extends beyond the initial hospitalization, as post-discharge care can play an important role in mitigating the burden associated with injury.^15–19^ Those who survive the inciting event remain at risk for complications, including in-hospital mortality and life-altering morbidity. The CDC estimates the combined cost of lost work for patients who are treated for injuries in the emergency department (ED) or hospitalized after trauma to be more than $250 billion.^1^ Functional recovery is a factor in determining the likelihood of returning to work after sustaining an injury, which makes an increase in access to rehabilitative services an important target for not only improving quality of life, but also mitigating the financial burden associated with trauma.^16,20,21^ Rehabilitative therapy can be used to mitigate the sequela of injury or to help with functional recovery after a prolonged hospital stay. Skilled nursing facilities can act as a bridge for patients in need of ongoing support after discharge, and home healthcare services provide adjunctive care that can make a safe transition to home possible for more patients. Two multistate studies examining the first 1 to 2 years after ACA Medicaid expansion implementation found expansion to be associated with a decrease in uninsured rates, an increase in access to rehabilitation, and reductions in racial and socioeconomic disparities in some outcomes among nonelderly adult trauma patients.^22,23^ Results from studies of individual Medicaid expansion states or trauma centers have indicated that although uninsured patients remain at an increased risk of mortality after trauma, the ACA Medicaid expansion has also led to a decrease in traumatic injury-related ED utilization and an increase in hospital revenue, particularly among safety net hospitals. Findings with regard to hospital length of stay among trauma patients have been mixed.^24–26^ To date, studies examining the effects of the ACA Medicaid expansion on access to care and outcomes among trauma patients have been limited by their examination of just the first 1 to 2 years of Medicaid expansion, their single state or single-center design, or their examination of in-hospital outcomes only.^27^

The aim of this study was to evaluate the effects of the ACA Medicaid expansion, in its first 4 years, on insurance coverage, trauma outcomes, and access to rehabilitation among young adult trauma patients across multiple states, overall and by race, ethnicity, and community income level. We hypothesized that the ACA Medicaid expansion has been associated with reductions in uninsurance, in-hospital mortality, and unplanned ED visits and readmissions at 30 and 90 days, and that it has increased access to rehabilitation. We also hypothesized that the ACA Medicaid expansion has been associated with decreases in racial, ethnic, and socioeconomic disparities in these outcomes.

## METHODS

### Data sources

Data came from the State Inpatient Databases (SIDs) of 5 Southern and Midwestern states that implemented the ACA Medicaid expansion in January 2014 (Arkansas, Illinois, Iowa, Kentucky, and Maryland) and 5 Southern and Midwestern states that had not yet adopted or implemented the ACA Medicaid expansion by the end of 2017 (Florida, Georgia, Kansas, Missouri, and North Carolina). These states were selected because they each have a young adult population that is at least 10% Black or Hispanic, had not implemented a state-wide expansion of insurance coverage to adults without dependents with incomes up to or above the federal poverty level before 2014 or implemented ACA Medicaid expansion in a delayed fashion, and had high-quality data on patient race and ethnicity (<10% missing) and either ZIP code-level median household income quartile or patient residential ZIP codes (<10% missing). These states were also selected because, together, the included Medicaid expansion and non-expansion states are similar in their geographic, sociodemographic, and trauma system characteristics (eTable 1). Data from 2011–2017 were used after being obtained from the AHRQ Healthcare Cost and Utilization Project central distributor or from state departments of health. The SIDs include all inpatient discharge records from all community hospitals in a state. The data are derived from discharge summaries and billing records.^31^ Data use agreements were completed with AHRQ, Illinois Department of Public Health, and Missouri Department of Health and Senior Services. The study was approved by the IRB of Illinois Department of Public Health and by our local IRB with a waiver of informed consent.

### Study population

This study included patients aged 19 to 44 years who were hospitalized for traumatic injury at an acute care community hospital in 1 of the 10 selected states and who were discharged between January 1, 2011 and December 31, 2017. To identify hospitalizations for traumatic injury, we followed the National Trauma Data Standard.^32^ Hospitalizations had to have a principal ICD-9-CM diagnosis code in the range from 800.x. to 959.x., excluding 905 to 909, 910 to 924, 930 to 939, or a principal ICD-10-CM diagnosis code of S00 to S99 (with seventh-character modifiers of A, B, or C), T07, T14, T20, to T28 (with seventh-character modifier of A), T30 to T32 or T79.A1 to T79.A9 (with seventh-character modifier of A), excluding S10, S20, S30, S40, S50, S60, S70, S80, or S90. Patients were also required to reside in the state in which they were hospitalized. The transferring hospital records of patients transferred to other short-term hospitals were excluded, with only the admission at the receiving hospital retained. Finally, patients with a burn injury as their primary diagnosis (ICD-9-CM 940 to 949; ICD-10-CM T20 to T32); patients admitted electively; and patients with missing data on discharge disposition, sex, race, ethnicity, national ZIP code-level median household income quartile, or urban or rural residence were excluded. Less than 3% of admissions were excluded due to having missing data on these key factors.

### Outcomes and covariates

Our outcomes of interest were insurance coverage (ie uninsured, Medicaid, private insurance, and other), in-hospital mortality, length of stay, discharge to rehabilitation (ie inpatient rehabilitation, skilled nursing facilities, or home healthcare), and any unplanned readmission or ED visit within 30 or 90 days. Unplanned readmissions were defined as nonelective readmissions and were examined only in states with patient identifiers (ie Arkansas, Florida, Georgia, Illinois, Iowa, and Maryland). Return ED visits were also examined in these states only. Unplanned readmissions and return ED visits were examined using data from 2013–2017 only, due to patient identifiers being absent in some states in earlier years. Year of discharge, state Medicaid expansion status, patient race and ethnicity, and patient residential ZIP code-level median household income quartile were the exposures of interest. However, other patient characteristics were evaluated and included in risk-adjusted outcomes models. These factors included patient age, sex, urban or rural residence, Injury Severity Score (ISS), presence of a severe head or neck injury, mechanism of injury, presence of any chronic condition, presence of traumatic shock, and whether an operative procedure was performed. These factors were selected for inclusion in multivariable analyses because of their known associations with at least 1 of the outcomes of interest. Presence of a severe injury, including a head or neck injury, presence of a comorbidity, and signs of shock have all been found to increase the risk of mortality after trauma, and operations introduce the risk of surgical complications and can be an indicator of injury severity.^33–37^ Patient residential urbanicity or rurality was included because geographic barriers affect patient access to trauma care. This was evaluated using the county-level scheme proposed by the National Center for Health Statistics.^38,39^ ISS was calculated using the ICD Program for Injury Categorization in R statistical software, version 0.1.0.^40^ The original ICD Program for Injury Categorization program was developed for use with ICD-9-CM codes, but ICD Program for Injury Categorization in R statistical software allows the use of both ICD-9-CM and ICD-10-CM codes, with options to calculate injury severity directly from ICD-10-CM codes (based on diagnosis-specific mortality estimates from the National Trauma Data Bank) or indirectly by first mapping ICD-10-CM codes to ICD-9-CM codes using the Centers for Medicare and Medicaid Services’ General Equivalence Mapping tables. We chose to use the General Equivalence Mapping minimum severity method for converting ICD-10-CM codes to ICD-9-CM codes because this strategy resulted in minimal disruption in trends across the coding transition.^41^ Mechanism of injury was defined by mapping diagnosis codes into categories according to the frameworks recommended by the CDC.^42–44^ Hospital characteristics, such as trauma center status and level, were not able to be considered because hospital identifiers that could be used for linkage to other databases with such characteristics were not available for Georgia, Kansas, or Missouri.

### Statistical analysis

We examined patient characteristics in the overall study cohort and in Medicaid expansion and non-expansion states before and after implementation of the ACA Medicaid expansion and open enrollment in January 2014, and we summarized these characteristics using frequencies and percentages, medians and interquartile ranges, or means and SDs. We then conducted difference-in-difference (DD) analyses to examine the impact of the ACA Medicaid expansion on insurance coverage and risk-adjusted outcomes. We first verified the parallel trends assumption required for DD analyses by comparing trends in outcomes in 2011–2013 between expansion and non-expansion states overall and within each racial or ethnic and ZIP code-level income group. No significant trends were detected overall or in any of the sociodemographic groups of interest. For the DD analyses, we used marginal logistic regression models for all binary outcomes and marginal negative binomial models for length of stay (LOS), fit using generalized estimating equations to account for patient clustering within hospitals. The models included time period (pre- or post-Medicaid expansion and open enrollment), state Medicaid expansion status, and their interaction. Models for insurance coverage did not contain additional covariates, but all other outcomes models included patient age, sex, urban or rural residence, ISS category (ie mild or moderate [ISS ≤ 15], severe [16 ≤ ISS ≤ 24], or extremely severe [ISS ≥ 25]), mechanism of injury based on the first listed external cause of injury code, whether a severe head or neck injury (head and neck Abbreviated Injury Scale score ≥ 3) was present, and whether the patient had any chronic conditions. Models for in-hospital mortality and LOS also included a covariate for whether the patient had experienced traumatic shock (ICD-9-CM 958.4; ICD-10-CM T794XXA). Models for unplanned readmissions and return ED visits also included a binary variable for whether a surgical procedure had been performed, as this is strongly associated with risk of readmission.^5,45,46^

To evaluate the impact of the ACA Medicaid expansion on racial, ethnic, and socioeconomic disparities in insurance coverage and risk-adjusted outcomes, we performed difference-in-difference-difference analyses using an analogous modeling approach. These models included the same covariates as described, but also included second-order interactions among time period, state Medicaid expansion status, and either patient race and ethnicity or the median national household income quartile of the patient’s residential ZIP code. All estimates of differences between Medicaid expansion and non-expansion states in changes in risk-adjusted outcomes from before to after Medicaid expansion and open enrollment were derived using marginal standardization either for the overall study cohort (in DD analyses) or within each sociodemographic group of interest (in difference-in-difference-difference analyses).^47,48^ We also conducted analyses in which DD and difference-in-difference-difference estimates were derived for each year after the implementation of ACA Medicaid expansion and open enrollment (2014–2017) compared with 2011–2013.

We repeated all analyses in the subgroup of patients with severe injuries (ISS > 15), as these patients were more likely to experience each of the outcomes of interest. We also repeated our analyses of the discharge to rehabilitation outcomes after restricting the study population to patients who had 1 or more of the presumptive diagnosis codes defined by the Centers for Medicare and Medicaid Services (CMS) as a requirement for financial support of inpatient rehabilitation facilities (CMS-funded inpatient rehabilitation facilities must support a case mix of patients comprising 60% or more of such codes, ie the 60% rule).^49^ Finally, we repeated all analyses after excluding the state of Maryland because Maryland hospitals substantially reduced their numbers of inpatient admissions for all conditions, including trauma, during the year before and the first year after the ACA Medicaid expansion (eFig. 1).^50^ All statistical analyses were performed using SAS Enterprise Guide, version 8.1 (SAS Institute). A p value < 0.05 was the threshold for statistical significance for all tests.

## RESULTS

### Patient characteristics

A total of 367,066 patients were included, 119,090 (32.4%) of whom were from ACA Medicaid expansion states. The majority of patients (73.3%) were male and mean (SD) age was 30.8 (7.6) years. Sociodemographic characteristics were largely similar between patients in the selected Medicaid expansion and non-expansion states, with Black patients comprising 27.0% and Hispanic patients comprising 13.6% of the total study population. However, patients from non-expansion states were more likely to reside in lower-income ZIP codes and patients from Medicaid expansion states were more likely to reside in higher-income ZIP codes. The 2 groups were similar with regard to their clinical and injury characteristics (Table 1). This was also the case in patient subgroups defined by race or ethnicity or ZIP code-level income quartile (eTables 2 through 8).

### Effects of the Affordable Care Act’s Medicaid expansion on insurance coverage

**Figure 1.**
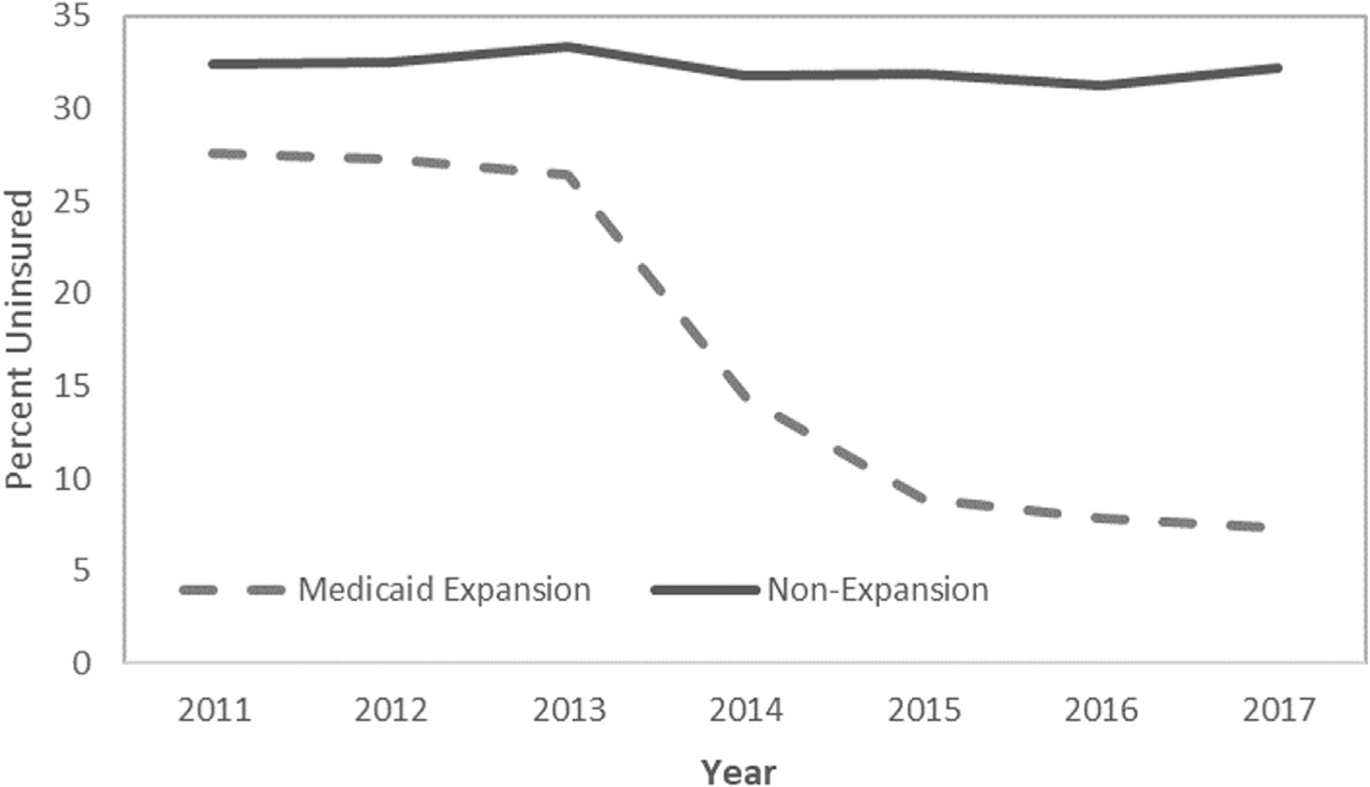
Proportion of young adult trauma patients uninsured over time, by state Medicaid expansion status.

**Figure 2.**
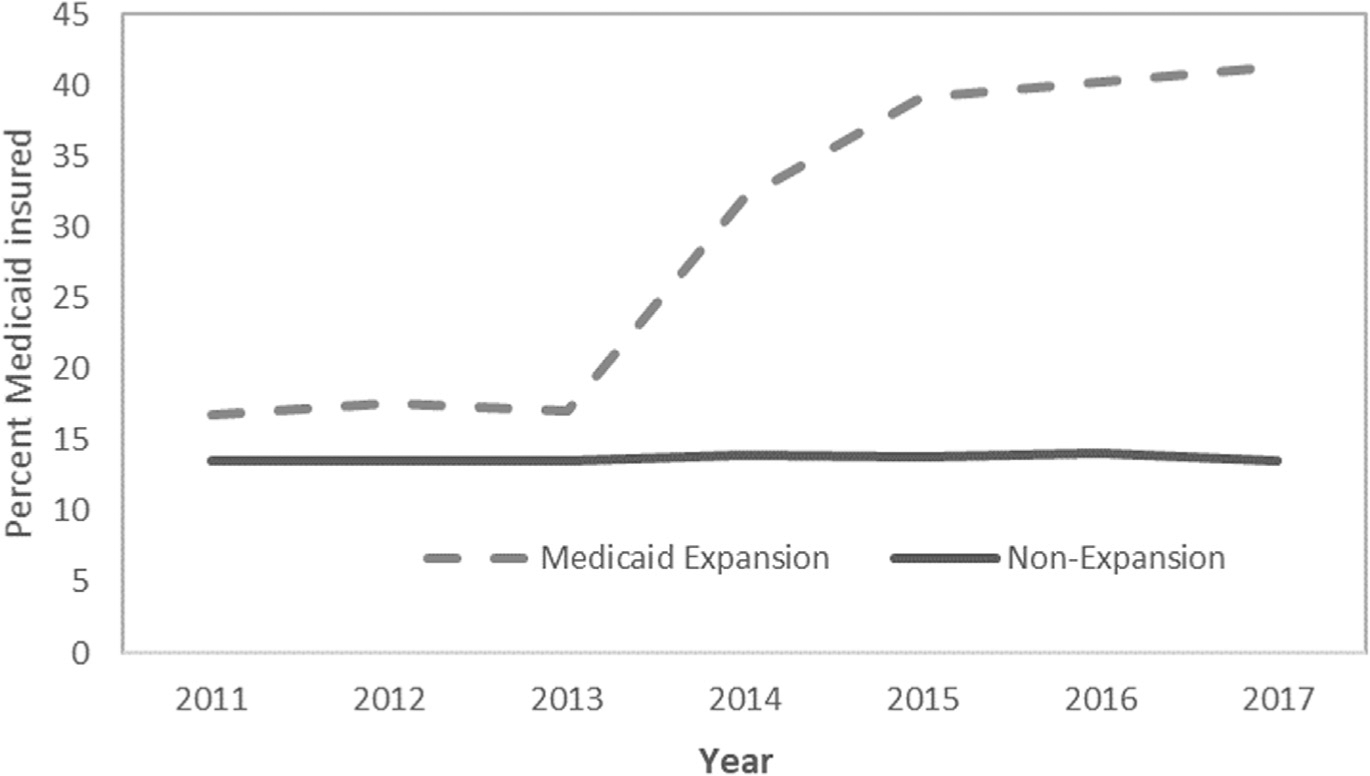
Proportion of young adult trauma patients covered by Medicaid over time, by state Medicaid expansion status.

**Figure 3.**
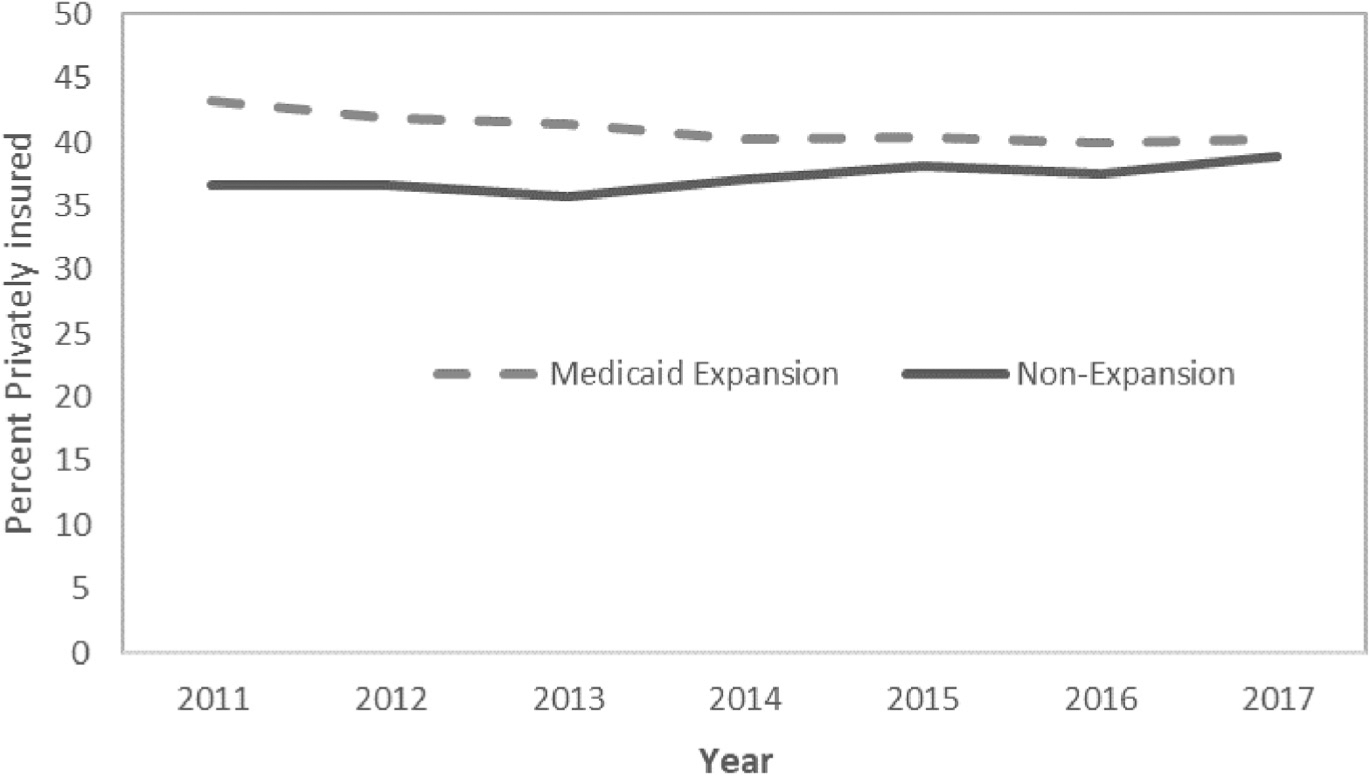
Proportion of young adult trauma patients covered by private insurance over time, by state Medicaid expansion status.

Implementation of the ACA Medicaid expansion was associated with a significant reduction in the uninsured rate, with this rate being 17.4 percentage points lower in 2014–2017 vs 2011–2013 in Medicaid expansion states, but only 0.9 percentage points lower in non-expansion states (DD −16.5 percentage points; 95% CI, −17.1 to −15.9 percentage points; p < 0.001) (Table 2). The uninsured rate decreased steadily over time post expansion in the Medicaid expansion states, but the reductions were greatest in 2014 and 2015 (Fig. 1). As expected, Medicaid expansion was associated with a significant increase in the proportion of young adult trauma patients covered by Medicaid, with Medicaid coverage increasing in Medicaid expansion states by 21.0 percentage points and increasing in non-expansion states by only 0.3 percentage points (DD 20.7 percentage points; 95% CI, 20.1 to 21.2 percentage points; p < 0.001). Medicaid coverage steadily increased over time post expansion in the Medicaid expansion states, but the increases were greatest in 2014 and 2015 (Fig. 2). The ACA Medicaid expansion was also associated with a significant reduction in the proportion of young adult trauma patients who had private insurance; private insurance coverage decreased in Medicaid expansion states by 2.0 percentage points and increased in non-expansion states by 1.5 percentage points (DD −3.5 percentage points; 95% CI, 4.2–2.8 percentage points; p < 0.001) (Fig. 3).

Medicaid expansion was associated with the greatest decreases in the likelihood of being uninsured among Black patients and patients from the lowest-income ZIP codes (Tables 3 and 4). Medicaid expansion was associated with a significant reduction in the likelihood of being uninsured among Hispanic patients, but the magnitude of this reduction was not as great as it was among White patients (Table 3). Medicaid expansion was associated with a reduction in the proportion of patients who were uninsured across all 4 quartiles of ZIP code-level median household income, but reductions were greater among patients who were not from the highest-income ZIP codes (Table 4). In contrast, the Medicaid expansion-associated reduction in private insurance coverage was largest among patients from the highest-income ZIP codes.

### Effects of the Affordable Care Act’s Medicaid expansion on trauma outcomes and access to rehabilitation

**Figure 4.**
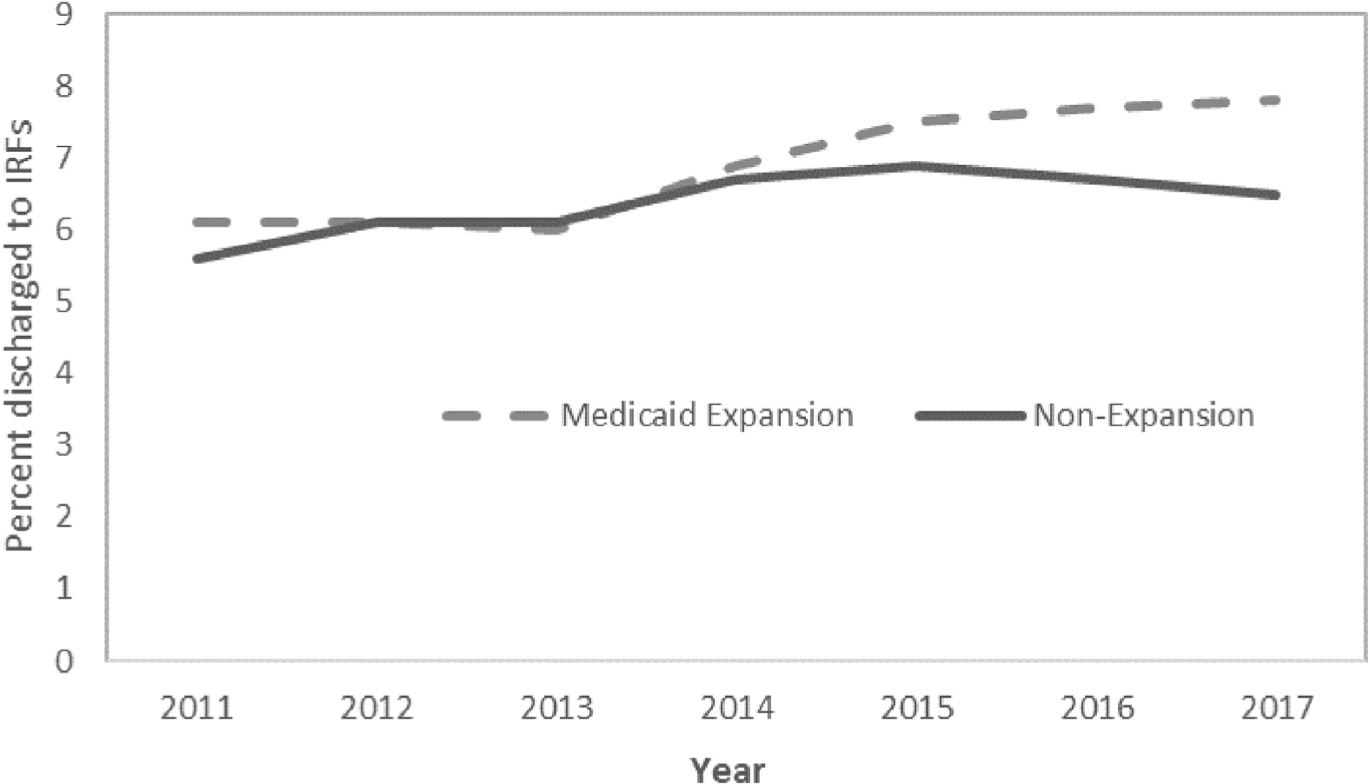
Proportion of young adult trauma patients discharged to inpatient rehabilitation facilities (IRFs) over time, by state Medicaid expansion status.

**Figure 5.**
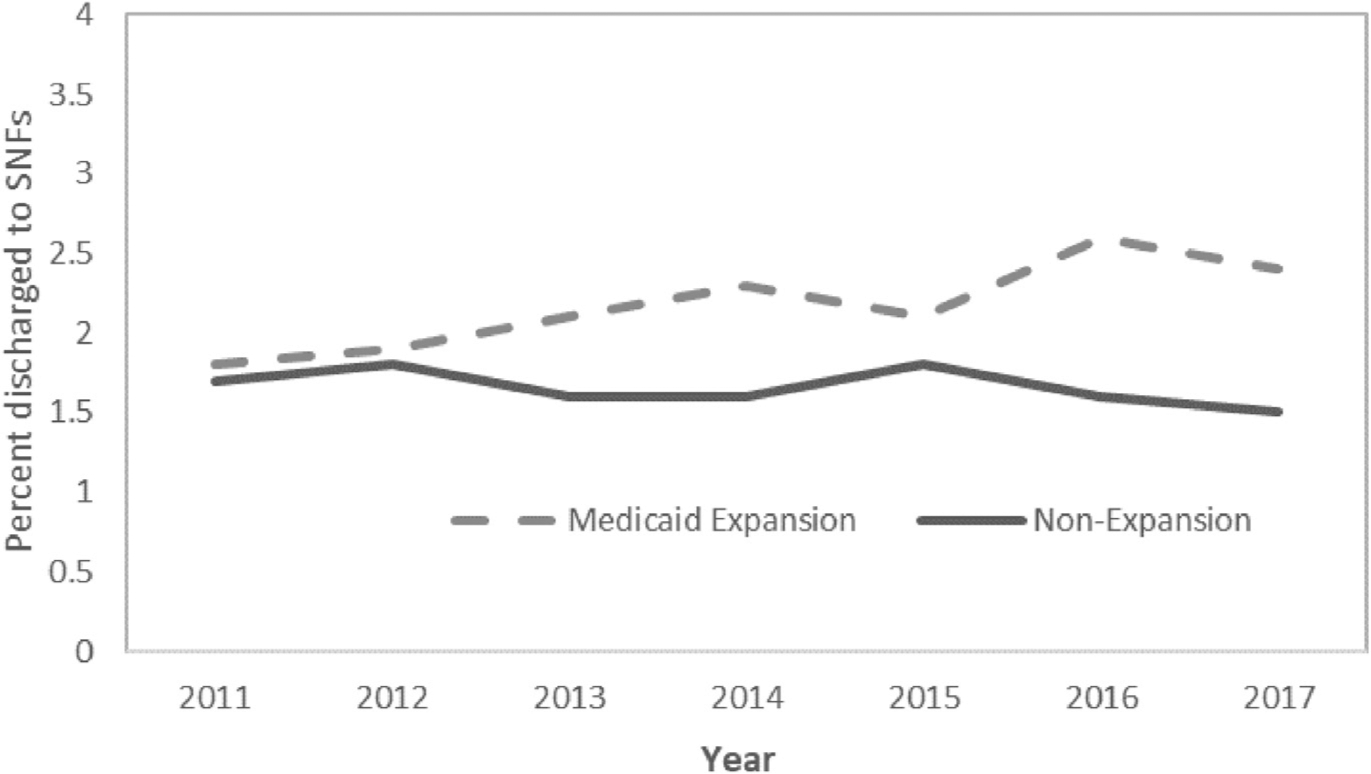
Proportion of young adult trauma patients discharged to skilled nursing facilities (SNFs) over time, by state Medicaid expansion status.

From 2011–2013 to 2014–2017, there was a slight reduction in in-hospital mortality in both the selected Medicaid expansion states and non-expansion states, and no significant difference in these reductions (DD −0.12 percentage points; 95% CI −0.3 to 0.05 percentage points; p = 0.17) (Table 2). Medicaid expansion was associated with a significant increase in risk-adjusted LOS (DD 0.3 days; 95% CI, 0.3 to 0.4 days; p < 0.001). It was also associated with an increase in the probability of being discharged to any rehabilitation, including significant increases in access to inpatient rehabilitation (DD 0.55 percentage points; 95% CI, 0.2 to 0.9 percentage points; p = 0.003) and to skilled nursing facilities (DD 0.50 percentage points; 95% CI 0.3 to 0.7 percentage points; p < 0.001). Access to inpatient rehabilitation increased steadily over time after Medicaid expansion in the selected Medicaid expansion states, although increases were greatest in 2014 and 2015 (Fig. 4). In contrast, the rate of discharge to skilled nursing facilities did not continually increase in all years after Medicaid expansion (Fig. 5). Medicaid expansion was not associated with changes in the proportion of patients with an unplanned readmission or ED visit within either 30 or 90 days after hospital discharge. However, there was an increase in the proportion of patients with a return ED visit within 90 days in both the Medicaid expansion and non-expansion states, with no difference between the 2 groups (DD 0.37 percentage points; 95% CI, −0.71 to 1.44 percentage points; p = 0.50) (Table 2).

When comparing outcomes by patient race and ethnicity, we found that Medicaid expansion was associated with a reduction in in-hospital mortality among Black patients, and this reduction was significantly greater than that seen among White patients. The decrease in in-hospital mortality among Black patients in expansion states was most prominent among patients with firearm injuries (DD −1.55 percentage points; 95% CI, −2.52 to −0.59 percentage points). In addition, the impact of Medicaid expansion on the proportion of young adult trauma patients discharged to any rehabilitation service was significantly greater among Black patients than White patients (Table 3). We found no significant differences between White and Hispanic patients in the impact of the ACA Medicaid expansion on any of the evaluated trauma outcomes. Medicaid expansion was associated with a greater increase in access to inpatient rehabilitation among patients from the lowest-income ZIP codes than among patients from the highest-income ZIP codes (Table 4). However, it was associated with a smaller increase in length of stay among patients from lower-income ZIP codes than among patients from the highest-income ZIP codes.

### Results of subgroup and sensitivity analyses

In the subgroup of patients with severe injuries (ISS > 15), Medicaid expansion was associated with a significant increase in access to inpatient rehabilitation (DD 1.44 percentage points; 95% CI, 0.02 to 2.86 percentage points; p = 0.04), an increase that was greater than that seen in the overall study cohort. Medicaid expansion was also associated with a decrease in the proportion of severely injured patients discharged to home health (DD −1.62 percentage points; 95% CI, −2.65 to −0.59 percentage points; p = 0.002). No other outcomes were found to be statistically significantly impacted by Medicaid expansion among severely injured patients (eTable 9). As was seen in the overall cohort, severely injured Black patients had a significantly greater Medicaid expansion-associated reduction in in-hospital mortality (DD −1.07 percentage points; 95% CI, −2.64 to 0.50 percentage points) than severely injured White patients (DD 0.67 percentage points; 95% CI, −0.32 to 1.67 percentage points) and also had a significantly greater Medicaid expansion-associated increase in discharge to any rehabilitation (DD 3.80 percentage points; 95% CI, 0.10 to 7.51 percentage points) than severely injured White patients (DD −0.78 percentage points; 95% CI, −2.88 to 1.31 percentage points) (eTable 10). In contrast, severely injured Hispanic patients experienced a greater Medicaid expansion-associated decrease in the likelihood of being discharged to home healthcare (DD −5.64 percentage points; 95% CI, −8.94 to −2.34 percentage points) than severely injured White patients (DD −1.68 percentage points; 95% CI, −2.91 to −0.46 percentage points). As was seen in the overall study cohort, the impact of Medicaid expansion on access to inpatient rehabilitation was greater among patients from the lowest-income ZIP codes (DD 3.31 percentage points; 95% CI, 1.11 to 5.51 percentage points) compared with the highest-income ZIP codes (DD −0.97 percentage points; 95% CI, −5.12 to 3.18 percentage points) (eTable 11).

In the subgroup of patients with 1 or more of the presumptive diagnosis codes defined by CMS as a requirement for financial support of inpatient rehabilitation facilities, Medicaid expansion was associated with increases in the proportion of patients discharged to any rehabilitation, to inpatient rehabilitation, and to a skilled nursing facility, and a decrease in the proportion of patients discharged to home healthcare (eTable 12). In this subgroup, there were no differences in the effects of Medicaid expansion by race and ethnicity, but Medicaid expansion was associated with a greater increase in the rate of discharge to inpatient rehabilitation among patients from the lowest-compared with highest-income ZIP codes. In contrast, Medicaid expansion was associated with a greater increase in the rate of discharge to skilled nursing facilities among patients from the highest-income ZIP codes compared with patients from lower-income ZIP codes (eTable 13).

Finally, we conducted analyses that excluded the state of Maryland because of its hospitals’ substantial reduction in trauma admissions from 2012 to 2014 (eFig. 1). All results were similar to those of the primary analysis, except for LOS. When patients from Maryland were excluded, Medicaid expansion was no longer associated with a change in LOS overall or among patients from higher- or lower-income ZIP codes (eTable 14). We also conducted sensitivity analyses to ensure that the significant association between Medicaid expansion and decreased in-hospital mortality among Black patients was not driven by a single state or year. We re-evaluated this end point in a series of sensitivity analyses, each excluding a single state (eTable 15). The magnitude of the Medicaid expansion-associated decrease in in-hospital mortality among Black patients was similar across all of these analyses. Additional analyses showed that Medicaid expansion-associated reductions in in-hospital mortality among Black patients were greatest in the first 2 years of Medicaid expansion, with rates remaining stable thereafter (eFig. 2).

## DISCUSSION

This study found that among young adult trauma patients, the ACA Medicaid expansion, in its first 4 years of implementation, was associated with a decrease in the proportion of patients who were uninsured and an increase in the proportion discharged to rehabilitative services. Medicaid expansion was not associated with a change in the rate of in-hospital mortality or rates of unplanned readmissions or return ED visits within 30 or 90 days. It was associated with an increase in hospital LOS. However, this increase in LOS was no longer present after excluding patients from Maryland, likely because, from 2012 to 2014, there was a large reduction in the number of inpatient trauma admissions among nonseverely injured patients in Maryland, and observation status encounters were not included in this study. Despite not being associated with a reduction in in-hospital mortality overall, the ACA Medicaid expansion was associated with a reduction in in-hospital mortality among Black patients. In addition, gains in insurance coverage and access to rehabilitation were larger among Black patients than White patients and also larger among patients from lower-income ZIP codes than higher-income ZIP codes. Overall, this suggests that Medicaid expansion has had a positive effect on young adult trauma patients by increasing insurance coverage and access to rehabilitative care, and by reducing racial and socioeconomic disparities in insurance coverage, access to rehabilitative care, and in-hospital mortality.

The largest gain in insurance coverage for trauma patients in our study occurred within the first 2 years of the ACA Medicaid expansion implementation, which largely explains why the increase in coverage after 4 years was not vastly different from that reported in previous studies that examined only the first 1 to 2 years of Medicaid expansion.^22,23,25,51–53^ At the end of 2017, 32% of young adult trauma patients treated in non-expansion states were uninsured compared with only 9.7% of patients treated in expansion states. However, racial and ethnic disparities in insurance coverage persist nationally and among the young adult trauma patients in the Medicaid expansion states in this study, with 21% of Hispanic trauma patients in the included expansion states remaining uninsured in 2017 compared with 9.4% of Black patients and 7.1% of White patients. In addition, although the socioeconomic disparity and the disparity between Black and White patients in insurance coverage decreased, the disparity between Hispanic and White patients in insurance coverage actually increased. These findings largely mirror those of studies comparing changes in insurance coverage among all nonelderly adults nationally, which have found that disparities in insurance coverage have persisted despite Medicaid expansion-associated decreases in uninsured rates that are greater among Black and Hispanic adults compared with White adults and among lower-income compared with higher-income adults.^14,54–57^ By expanding our study beyond the first 1 to 2 years of ACA Medicaid expansion implementation, we were able to identify a slowing of the trend toward a reduced uninsured rate among trauma patients, as well as a persistence of racial and ethnic disparities in this rate. Hispanic patients are more likely to be affected by the current policy that makes undocumented residents and legal permanent residents who have resided in the US for fewer than 5 years ineligible for Medicaid coverage, but this alone is insufficient to fully explain the persistent gap between Hispanic and non-Hispanic White patients, and suggests the presence of other systemic barriers to health insurance coverage.^58^ Nevertheless, making Medicaid or ACA Marketplace plans available to undocumented and recent immigrants would help close the coverage gap, as would outreach to encourage and facilitate enrollment in coverage among eligible Hispanic young adults. In contrast, public charge rule changes and other immigration-related policies that caused many families to turn away from Medicaid coverage and health-care utilization in recent years likely also negatively impacted coverage and access to care disproportionately among Hispanic young adult trauma patients.

This study found that, among young adult trauma patients, the ACA Medicaid expansion led to a significant increase in hospital LOS and greater access to facility-based rehabilitative care, but not home healthcare. A study by Zogg and colleagues^23^ that examined the effect of the ACA Medicaid expansion in its first 2 years across 9 states reported an increase in hospital LOS, as well as an increase in access to all forms of rehabilitation, including home healthcare. The observed increase in LOS in our study was negated after removing Maryland from the analysis, mirroring previous studies that have found that changes in LOS among trauma patients after Medicaid expansion varied by state and injury severity.^8,25,59,60^ The paucity of post-discharge care facilities in some regions can create a bottleneck for patients awaiting discharge to these specialized services and, when combined with variation across states and hospitals over time in decision making about admitting vs observing nonseverely injured patients, contributes to variability in LOS among admitted trauma patients.^61^ In contrast, the difference in reported access to home healthcare is likely a result of study cohort selection. Home healthcare is typically reserved for patients in need of assistance with wound care or activities of daily living. Because the study by Zogg and colleagues included all nonelderly adult trauma patients, patients in that study were considerably older on average (mean 41.9 vs 30.8 years) and more likely to need home healthcare at the time of discharge. Notably, in our study there was a larger increase in access to inpatient rehabilitation among the patients most likely to experience functional impairment after trauma, including those with severe injuries and those meeting CMS criteria for inpatient rehabilitation. Among all young adult trauma patients, there were no differences between Hispanic and White patients in their gains in access to rehabilitative care. However, among severely injured patients, there was a greater decrease in the proportion of patients discharged to home healthcare among Hispanic patients than White patients. This suggests that the smaller health insurance coverage gains for Hispanic patients might have been compounded by language and cultural barriers to home healthcare access, as studies have shown that patients with limited English proficiency are generally less likely to receive referrals to, and to seek, medical care.^62–64^ This finding highlights the need for policies and initiatives that improve healthcare providers’ cultural and language competency. In particular, policies that increase the availability of on-site interpreters at rehabilitation facilities and that promote the hiring, development, and retention of culturally competent rehabilitation and home healthcare workers can reduce the underuse of these types of care among Hispanic young adult trauma patients. The significant increase in ED visits at 90 days in both Medicaid expansion and non-expansion states and the lack of an association between Medicaid expansion and ED utilization were somewhat surprising, given the known association between uninsurance and greater ED utilization and previous study findings of an association between the ACA Medicaid expansion and decreased population-level injury-related ED utilization.^59^ However, the results of other studies examining the impact of the ACA Medicaid expansion on overall ED utilization in the general population or specific subpopulations (eg low-income adults) have been mixed.^56^ The greater availability of emergency care in expansion states compared with non-expansion states is also worth noting.^65^ However, barriers that patients experience in connecting with outpatient services post discharge and deficiencies in the quality of hospital care also affect the likelihood of a patient returning to the ED after discharge.^66,67^ Importantly, although the ACA Medicaid expansion has been associated with reductions in hospitals’ uncompensated care and higher operating margins, it is not yet clear whether this has resulted in improved quality of care.^68–70^ Managing the unique needs of trauma patients is challenging, and for patients who do not meet criteria for formal post-discharge services, it is essential to create reasonable expectations about at-home recovery and to establish connections with outpatient providers before the patient leaves the hospital. An emphasis on decreasing regional variability in access to quality post-discharge services could improve healthcare outcomes and decrease the cost of care by minimizing inefficiencies in the current system.^61^

We hypothesized that the ACA Medicaid expansion would be associated with a reduction in in-hospital mortality among young adult trauma patients, given the previously identified link between being uninsured and having a greater risk of mortality after trauma.^4^ It remains unclear why this study and previous studies have failed to identify an association between an increase in insurance coverage and in-hospital mortality among trauma patients, but the low in-hospital mortality rate among young adults might partly explain this finding. The majority of young adults in this study had 1 or no documented comorbidities, and our examination of only a 4-year time period post expansion might have also limited the potential of this study to detect a survival benefit related to an increase in patients’ access to pre-injury primary and preventive healthcare.^35^ Implementation of the ACA has led to an increase in access to medications and increases in the use of primary care and preventative visits, but the impact of these changes on the population-level health of young adults is not fully understood.^53,57,71–73^ It is also important to note that being uninsured is correlated with numerous other factors that independently affect trauma outcomes, such as treatment delay, provider race and social class biases, and treatment at lower-resourced hospitals, and some of these factors are unlikely to have been impacted by the ACA Medicaid expansion. Importantly, across patients with any injury severity and patients with the most severe injuries, Medicaid expansion was associated with a significant reduction in in-hospital mortality among Black patients. Although the reasons for this finding are unclear, the decrease in in-hospital mortality among Black patients in expansion states was most prominent among patients with firearm injuries. This suggests that mechanisms for the decrease in in-hospital mortality among Black patients might include improved access to high-quality prehospital care, more timely access to definitive care, or improved inpatient care quality. We were unable to test these mechanisms in the current study, but other studies have demonstrated that safety net hospital and public hospitals, which often serve large numbers of Black patients, have experienced the greatest Medicaid expansion-associated gains in resources.^74^ Whether this has led to improvements in the quality of care, however, remains unclear. Despite their mortality reduction, Black patients remained more likely to experience in-hospital mortality than White patients after Medicaid expansion. Racial differences in factors that were not available in this study, such as detailed anatomic and physiologic injury characteristics and provider and hospital characteristics, might explain this persistent disparity.^75–77^

This study had several limitations, most of which result from its reliance on administrative hospital discharge data. With such data, there is always the potential for misclassified and incomplete data. We attempted to minimize this by selecting states with high-quality data on the key variables of interest and by making use of validated code sets to define our study population and to define injury characteristics. The use of the SIDs also precludes the identification of some important patient clinical characteristics that affect mortality and other trauma outcomes, including physiologic markers. This was a necessary tradeoff made to allow the inclusion of all trauma-related hospitalizations rather than only those at trauma centers, as are found in most state trauma registries. Unfortunately, because several states’ databases lacked hospital identifiers, we also could not examine hospital characteristics, which play an important role in trauma outcomes. We assumed that such characteristics changed minimally in the selected states during the study period. Importantly, the study period included the transition from the ICD-9-CM to ICD-10-CM coding system, which might have created inconsistencies in the recording of trauma-related injuries and, as a result, inconsistencies in some covariates, such as ISS and mechanisms of injury. To evaluate and minimize this, we previously conducted a study examining the impact of the coding transition on trends in trauma admissions among young adults by injury mechanism, type, and severity, and the results of that study informed our selection of covariates for the current study.^41^ Another limitation of this study was the fact that, because patient identification numbers in the databases used are state-specific, information on readmissions and return ED visits that occurred in states other than the state in which the patient was originally hospitalized was not available. Finally, this study included only 10 states. We selected these states for the quality of their data and to ensure similarity of the Medicaid expansion and non-expansion cohorts with regard to geography, patient sociodemographic characteristics, and trauma system characteristics. Future studies should evaluate the effects of the ACA Medicaid expansion on trauma care and outcomes nationally, as well as examine its effects on long-term health, functional, and economic outcomes in trauma patients.

## CONCLUSIONS

This was the first study of the impact of the ACA Medicaid expansion on trauma care and outcomes that included data from more than the first 1 to 2 years of Medicaid expansion and that evaluated outcomes after hospital discharge. The study found that the ACA Medicaid expansion, in its first 4 years, led to increases in insurance coverage and access to rehabilitative services among young adult trauma patients. However, the ACA Medicaid expansion had no impact on readmission or return ED visit rates in this population. Medicaid expansion was associated with a significant reduction in in-hospital mortality among Black trauma patients, but not among White or Hispanic patients. Medicaid expansion was also associated with a reduction in the disparity in access to rehabilitative care between Black and White patients and between patients from lower- and higher-income communities. Based on these findings, Medicaid expansion has led to between 5 and 81 fewer in-hospital deaths per 10,000 Black young adult trauma patients and between 64 and 174 more admissions to inpatient rehabilitation facilities per 10,000 young adults who have complex injuries and who reside in low-income communities. Medicaid expansion has likely also led to overall improvements and reductions in disparities in trauma patients’ economic stability and long-term quality of life and functional outcomes. However, future studies examining the long-term effects of the ACA Medicaid expansion on trauma care and outcomes and on racial, ethnic, and socioeconomic disparities in trauma care and outcomes are warranted.

## Supplementary Material

1

## Figures and Tables

**Table 1.** Characteristics of Young Adult Trauma Patients in the Selected Medicaid Expansion and Non-Expansion States

Characteristic	Medicaid expansion (n = 119,090)	Non-Medicaid expansion (n = 247,976)	Total (n = 367,066)
Age, y, mean (SD)	30.7 (7.6)	30.8 (7.5)	30.8 (7.6)
Sex, n (%)			
Male	87,588 (73.5)	181,539 (73.2)	269,127 (73.3)
Female	31,502 (26.5)	66,437 (26.8)	97,939 (26.7)
Race and ethnicity, n (%)			
Non-Hispanic White	66,928 (56.2)	135,086 (54.5)	202,014 (55.0)
Non-Hispanic Black	32,502 (27.3)	66,457 (26.8)	98,959 (27.0)
Hispanic (any race)	13,201 (11.1)	36,898 (14.9)	50,099 (13.6)
Other	6,459 (5.4)	9,535 (3.8)	15,994 (4.4)
ZIP code-level median household income quartile, n (%)			
Quartile 1 (lowest)	41,575 (34.9)	106,347 (42.9)	147,922 (40.3)
Quartile 2	23,958 (20.1)	75,896 (30.6)	99,854 (27.2)
Quartile 3	28,136 (23.6)	46,625 (18.8)	74,761 (20.4)
Quartile 4 (highest)	25,421 (21.3)	19,108 (7.7)	44,529 (12.1)
Primary payer, n (%) (n = 365,837)			
Medicare	4,450 (3.8)	8,212 (3.3)	12,662 (3.5)
Medicaid	33,420 (28.2)	33,841 (13.7)	67,261 (18.4)
Private insurance	48,759 (41.1)	92,130 (37.3)	140,889 (38.5)
Self-pay	19,852 (16.7)	70,072 (28.3)	89,924 (24.6)
No charge	1,398 (1.2)	9,451 (3.8)	10,849 (3.0)
Other	10,670 (9.0)	33,582 (13.6)	44,252 (12.1)
Urban or rural residence, n (%)			
Large central metropolitan (>1 million population)	36,916 (31.0)	69,577 (28.1)	106,493 (29.0)
Large fringe metropolitan (>1 million population)	29,473 (24.7)	70,162 (28.3)	99,635 (27.1)
Medium metropolitan (250,000–999,999 population)	17,797 (14.9)	53,378 (21.5)	71,175 (19.4)
Small metropolitan (50,000–249,999 population)	11,258 (9.5)	19,676 (7.9)	30,934 (8.4)
Micropolitan	12,064 (10.1)	20,984 (8.5)	33,048 (9.0)
Noncore	11,582 (9.7)	14,199 (5.7)	25,781 (7.0)
No. of chronic conditions, n (%)			
0	32,476 (27.3)	76,344 (30.8)	108,820 (29.6)
1	30,829 (25.9)	66,015 (26.6)	96,844 (26.4)
2	21,751 (18.3)	43,270 (17.4)	65,021 (17.7)
3	13,675 (11.5)	26,401 (10.6)	40,076 (10.9)
> 3	20,359 (17.1)	35,946 (14.5)	56,305 (15.3)
Operative procedure, n (%)	67,239 (56.5)	149,410 (60.3)	216,649 (59.0)
Traumatic shock, n (%)	2,974 (2.5)	6,733 (2.7)	9,707 (2.6)
Injury Severity Score, n (%) (n = 367,039)			
Mild or moderate (0–15)	96,014 (80.6)	199,695 (80.5)	295,709 (80.6)
Severe (16–24)	16,226 (13.6)	34,144 (13.8)	50,370 (13.7)
Extremely severe (25–75)	6,839 (5.7)	14,121 (5.7)	20,960 (5.7)
Severe head or neck injury, n (%)	20,890 (17.5)	41,266 (16.6)	62,156 (16.9)
Injury mechanism, n (%)			
Cut or pierce	8,939 (7.5)	16,996 (6.9)	25,935 (7.1)
Fall	24,204 (20.3)	45,523 (18.4)	69,727 (19.0)
Firearm	12,367 (10.4)	22,579 (9.1)	34,946 (9.5)
Motor vehicle traffic	37,256 (31.3)	92,183 (37.2)	129,439 (35.3)
Struck by or against	10,439 (8.8)	19,413 (7.8)	29,852 (8.1)
Injury intent, n (%)			
Undetermined or unintentional	85,545 (71.8)	194,999 (78.6)	280,544 (76.4)
Self-harm	2,488 (2.1)	5,385 (2.2)	7,873 (2.1)
Assault	23,586 (19.8)	38,542 (15.5)	62,128 (16.9)

**Table 2.** Insurance Coverage and Risk-Adjusted Outcomes among Young Adult Trauma Patients in the Selected Medicaid Expansion and Non-Expansion States

	Expansion state		Non-expansion state			
Insurance coverage and outcomes	2011–2013 (n = 56,196)	2014–2017 (n = 62,894)	2011–2013 (n = 104,348)	2014–2017 (n = 143,628)	Difference-in-difference estimate (95% CI)	p Value
Uninsured	27.1	9.7	32.7	31.8	−16.50 (−17.08 to −15.93)	< 0.001*
Medicaid	17.1	38.1^†^	13.5	13.8	20.69 (20.13 to 21.25)	< 0.001*
Private insurance	42.2	40.2^†^	36.4	37.9^†^	−3.48 (−4.16 to −2.80)	< 0.001*
In-hospital mortality	2.2	1.9^†^	2.0	1.9^†^	−0.12 (−0.30 to 0.05)	0.17
Length of stay, d, mean	4.7	5.1^†^	5.5	5.6^†^	0.32 (0.25 to 0.39)	< 0.001*
Discharged to any rehabilitation	15.8	17.4^†^	15.4	16.1^†^	0.95 (0.40 to 1.50)	0.001*
Discharged to inpatient rehabilitation	6.1	7.4^†^	5.9	6.7^†^	0.55 (0.19 to 0.91)	0.003*
Discharged to a skilled nursing facility	1.9	2.3^†^	1.7	1.6	0.50 (0.29 to 0.72)	< 0.001*
Discharged to home healthcare	7.8	7.6	7.8	7.7	−0.16 (−0.58 to 0.25)	0.43
30-d unplanned readmission	7.5	8.0	7.9	8.1	0.34 (−0.32 to 1.00)	0.31
30-d return ED visit	17.6	18.3	18.4	19.2	−0.19 (−1.14 to 0.76)	0.69
90-d unplanned readmission	10.4	10.9	11.0	11.1	0.44 (−0.32 to 1.21)	0.25
90-d return ED visit	25.3	27.0^†^	25.8	27.2^†^	0.37 (−0.71 to 1.44)	0.50

Values are risk-adjusted marginal percentages unless otherwise indicated.

* Statistically significant.

† p < 0.05 vs 2011–2013 in the same states. For all trauma outcomes models, C statistics were > 0.65 and Hosmer-Lemeshow goodness-of-fit tests yielded p values > 0.05.

ED, emergency department.

**Table 3.** Insurance Coverage and Risk-Adjusted Outcomes among Young Adult Trauma Patients in the Selected Medicaid Expansion and Non-Expansion States: Results by Race or Ethnicity

	Expansion state		Non-Expansion state		
Insurance coverage and outcomes by race and ethnicity	2011–2013 (n = 56,196)	2014–2017 (n = 62,894	2011–2013 (n = 104,348	2014–2017 (n = 143,628)	Difference-in-differences estimate (95% CI)
Uninsured					
White	22.4	7.1	28.0	26.7	−13.91 (−14.62 to −13.19)
Black or African American	33.7	9.4	40.1	39.0	−23.13 (−24.29 to −21.97)*
Hispanic	35.9	21.4	38.9	36.0	−11.59 (−13.43 to −9.74)*
Medicaid					
White	13.7	32.1	12.3	12.1	18.57 (17.86–19.28)
Black or African American	26.4	55.2	16.6	16.9	28.50 (27.32–29.67)*
Hispanic	13.4	29.0	13.5	14.6	14.44 (12.89–15.98)*
Private insurance					
White	50.0	47.4	42.9	45.3	−4.99 (−5.91 to −4.06)
Black or African American	27.2	26.7	24.7	26.9	−2.74 (−3.92 to −1.56)*
Hispanic	35.8	36.5	30.0	32.1	−1.42 (−3.33 to 0.49)*
In-hospital mortality					
White	1.9	1.8	1.9	1.8	−0.02 (−0.24 to 0.20)
Black or African American	3.1	2.4	2.3	2.0	−0.43 (−0.81 to −0.05)*
Hispanic	1.5	1.2	1.8	1.5	−0.09 (−0.52 to 0.34)
Length of stay					
White	4.6	4.9	5.3	5.4	0.27 (0.18 to 0.36)
Black or African American	4.9	5.5	5.9	6.0	0.42 (0.28 to 0.55)
Hispanic	4.6	4.9	5.4	5.3	0.41 (0.22 to 0.59)
Discharged to any rehabilitation					
White	16.5	18.0	16.8	17.8	0.46 (−0.27 to 1.19)
Black or African American	15.3	17.4	14.0	14.4	1.77 (0.61 to 2.94)*
Hispanic	15.0	16.1	12.3	12.8	0.61 (−0.87 to 2.09)
Discharged to inpatient rehabilitation					
White	6.8	8.2	6.6	7.5	0.49 (−0.003 to 0.98)
Black or African American	5.2	6.8	5.1	5.9	0.80 (0.06 to 1.55)
Hispanic	4.4	5.5	4.7	5.2	0.50 (−0.39 to 1.38)
Discharged to a skilled nursing facility					
White	2.0	2.4	1.9	1.9	0.41 (0.12 to 0.70)
Black or African American	1.9	2.4	1.7	1.5	0.61 (0.16 to 1.07)
Hispanic	1.6	2.0	1.0	0.9	0.44 (−0.10 to 0.98)
Discharged to home healthcare					
White	7.7	7.3	8.3	8.4	−0.51 (−1.05 to 0.02)
Black or African American	8.1	8.2	7.3	7.0	0.39 (−0.51 to 1.28)
Hispanic	9.0	8.7	6.6	6.6	−0.33 (−1.53 to 0.87)
30-d unplanned readmission					
White	7.6	7.9	8.4	8.4	0.23 (−0.69 to 1.16)
Black or African American	8.2	9.3	7.4	8.2	0.29 (−0.95 to 1.52)
Hispanic	5.2	6.1	7.3	7.3	0.89 (−0.73 to 2.50)
30-d return ED visit					
White	16.7	17.0	17.8	18.9	−0.74 (−2.02 to 0.55)
Black or African American	20.9	22.2	21.5	22.0	0.62 (−1.23 to 2.47)
Hispanic	14.8	15.1	15.6	16.3	−0.32 (−2.77 to 2.13)
90-d unplanned readmission					
White	10.8	11.1	11.9	11.5	0.61 (−0.47 to 1.69)
Black or African American	11.4	12.0	10.6	11.0	0.23 (−1.19 to 1.65)
Hispanic	7.4	8.4	9.3	9.8	0.58 (−1.29 to 2.45)
90-d return ED visit					
White	24.6	25.6	25.6	26.9	−0.34 (−1.81 to 1.14)
Black or African American	30.2	32.5	29.0	30.8	0.50 (−1.57 to 2.56)
Hispanic	18.9	21.6	22.1	23.0	1.84 (−0.90 to 4.59)

Values are risk-adjusted marginal percentages.

* p < 0.05 vs difference-in-difference in non-Hispanic White patients. For all trauma outcomes models, C statistics were > 0.65 and Hosmer-Lemeshow goodness-of-fit tests yielded p values > 0.05.

ED, emergency department.

**Table 4.** Insurance Coverage and Risk-Adjusted Outcomes among Young Adult Trauma Patients in the Selected Medicaid Expansion and Non-Expansion States: Results by ZIP Code-Level Median Household Income Quartile

	Expansion state		Non-Expansion state		
Insurance coverage and outcomes by income quartile	2011–2013 (n = 56,196)	2014–2017 (n = 62,894	2011–2013 (n = 104,348	2014–2017 (n = 143,628)	Difference-in-differences estimate (95% CI)
Uninsured					
Quartile 1 (lowest)	34.2	10.1	37.6	36.1	−22.57 (−23.55 to −21.59)*
Quartile 2	27.7	10.5	32.3	30.7	−15.59 (−16.79 to −14.40)*
Quartile 3	25.2	9.8	28.4	26.5	−13.50 (−14.70 to −12.30)*
Quartile 4 (highest)	18.1	7.9	22.6	20.6	−8.18 (−9.61 to −6.75)
Medicaid					
Quartile 1 (lowest)	21.1	48.3	16.3	16.2	27.31 (26.32 to 28.29)*
Quartile 2	17.3	37.3	13.8	13.4	20.35 (19.15 to 21.56)*
Quartile 3	16.2	34.2	10.5	10.5	18.05 (16.91 to 19.18)*
Quartile 4 (highest)	12.1	25.6	7.5	7.6	13.42 (12.21 to 14.62)
Private insurance					
Quartile 1 (lowest)	29.4	29.7	28.2	30.6	−2.21 (−3.25 to −1.16)*
Quartile 2	40.0	39.1	36.5	39.6	−4.08 (−5.51 to −2.65)
Quartile 3	46.1	43.8	44.1	46.5	−4.67 (−6.14 to −3.19)
Quartile 4 (highest)	59.0	55.5	54.2	56.8	−6.13 (−7.99 to −4.26)
In-hospital mortality					
Quartile 1 (lowest)	2.36	2.17	2.14	1.96	−0.02 (−0.32 to 0.28)
Quartile 2	2.39	1.86	1.99	1.83	−0.36 (−0.75 to 0.01)
Quartile 3	1.94	1.82	1.99	1.85	0.02 (−0.35 to 0.39)
Quartile 4 (highest)	1.83	1.42	1.75	1.55	−0.21 (−0.64 to 0.23)
Length of stay					
Quartile 1 (lowest)	4.9	5.2	5.6	5.7	0.16 (0.05 to 0.27)*
Quartile 2	4.8	5.1	5.4	5.5	0.11 (−0.03 to 0.25)*
Quartile 3	4.6	5.1	5.4	5.4	0.50 (0.36 to 0.64)
Quartile 4 (highest)	4.2	4.9	5.4	5.4	0.56 (0.38 to 0.73)
Discharged to any rehabilitation					
Quartile 1 (lowest)	14.0	16.0	14.2	14.7	1.45 (0.61 to 2.30)
Quartile 2	16.2	17.2	16.0	16.5	0.50 (−0.62 to 1.62)
Quartile 3	17.0	19.0	16.0	17.2	0.69 (−0.53 to 1.91)
Quartile 4 (highest)	19.1	20.5	17.8	18.5	0.68 (−0.99 to 2.34)
Discharged to inpatient rehabilitation					
Quartile 1 (lowest)	5.2	7.2	5.2	6.0	1.19 (0.64 to 1.74)*
Quartile 2	6.7	7.4	6.2	6.9	−0.08 (−0.83 to 0.66)
Quartile 3	6.5	7.8	6.4	7.2	0.56 (−0.25 to 1.36)
Quartile 4 (highest)	7.2	8.1	7.1	8.2	−0.28 (−1.38 to 0.83)
Discharged to a skilled nursing facility					
Quartile 1 (lowest)	1.5	1.7	1.7	1.6	0.26 (−0.05 to 0.57)
Quartile 2	2.0	2.5	1.8	1.6	0.60 (0.14 to 1.05)
Quartile 3	2.2	2.8	1.7	1.8	0.56 (0.07 to 1.04)
Quartile 4 (highest)	2.4	3.2	1.6	1.5	0.96 (0.31 to 1.60)
Discharged to home healthcare					
Quartile 1 (lowest)	7.3	7.0	7.3	7.1	−0.03 (−0.66 to 0.61)
Quartile 2	7.5	7.3	8.1	8.0	−0.14 (−0.97 to 0.69)
Quartile 3	8.3	8.3	7.8	8.3	−0.45 (−1.36 to 0.46)
Quartile 4 (highest)	9.6	9.2	9.1	8.7	−0.09 (−1.35 to 1.17)
30-d unplanned readmission					
Quartile 1 (lowest)	7.6	7.8	7.7	8.3	−0.36 (−1.50 to 0.78)
Quartile 2	6.7	7.8	8.0	7.3	1.69 (0.31 to 3.06)
Quartile 3	6.8	7.9	7.4	8.4	0.12 (−1.23 to 1.47)
Quartile 4 (highest)	9.2	9.3	9.3	8.9	0.58 (−1.49 to 2.66)
30-d return ED visit					
Quartile 1 (lowest)	20.2	21.1	20.5	21.7	−0.35 (−2.05 to 1.34)
Quartile 2	17.5	18.1	19.0	19.4	0.11 (−1.88 to 2.10)
Quartile 3	15.7	16.3	16.6	17.3	−0.06 (−1.96 to 1.84)
Quartile 4 (highest)	13.0	13.5	13.7	14.7	−0.60 (−3.04 to 1.85)
90-d unplanned readmission					
Quartile 1 (lowest)	11.0	10.7	10.8	11.3	−0.82 (−2.15 to 0.51)
Quartile 2	9.6	11.0	11.2	10.3	2.32 (0.71 to 3.92)
Quartile 3	9.8	10.8	10.5	11.1	0.34 (−1.24 to 1.91)
Quartile 4 (highest)	11.4	11.6	12.3	11.7	0.78 (−1.54 to 3.10)
90-d return ED visit					
Quartile 1 (lowest)	28.5	30.7	28.5	30.7	−0.001 (−1.92 to 1.90)
Quartile 2	24.3	26.8	27.0	27.5	2.05 (−0.21 to 4.30)
Quartile 3	23.8	24.7	23.1	24.4	−0.51 (−2.69 to 1.68)
Quartile 4 (highest)	19.0	20.4	20.1	21.1	0.46 (−2.37 to 3.30)

Values are risk-adjusted marginal percentages.

* p < 0.05 vs difference-in-difference in highest income quartile. For all trauma outcome models, C statistics were > 0.65 and Hosmer-Lemeshow goodness-of-fit tests yielded p values > 0.05.

ED, emergency department.

## Abbreviations and Acronyms

ACA: Affordable Care Act
CMS: Centers for Medicare and Medicaid Services
DD: difference-in-difference
ED: emergency department
ISS: Injury Severity Score
LOS: length of stay
SID: State Inpatient Database
