# Supplementary material for: Association of the Affordable Care Act Medicaid Expansion with Trauma Outcomes and Access to Rehabilitation among Young Adults: Findings Overall, by Race and Ethnicity, and Community Income Level
Source: J Am Coll Surg. Author manuscript; Available in PMC 2021 Dec 1. (PMC8627499; doi:10.1016/j.jamcollsurg.2021.08.694)
Supplement: 1 [file NIHMS1742544-supplement-1.pdf]

eTable 1. Population and Trauma System Characteristics of the 5 Selected Medicaid Expansion and 5 Selected Non-Expansion States in 2013

Characteristic	Expansion	Non-expansion
Resident aged 18−44 y		
Uninsured, %	21	28
Non-Hispanic Black race or Hispanic ethnicity, %	31	38
Income below federal poverty level, %	17	18
Rural and urban, %		
Large central metropolitan	27	25
Large fringe metropolitan	35	29
Medium metropolitan	17	22
Small metropolitan	11	10
Micropolitan/noncore (rural)	20	15
Bachelor's degree or higher, %	37	30
Trauma system		
Resident within 60 min of Level I or II trauma center, %	86	93
Level I or II trauma centers per 1 million residents, n	1.9	1.2
Emergency department per 1 million residents, n	17.0	13.0
State uses CDC 2011 field triage guidelines, n/N	3/5	2/5
State has trauma destination bypass protocol in place, n/N	4/5	4/5

Data sources.[28-30]

eTable 2. Characteristics of Non-Hispanic White Young Adult Trauma Patients in the Selected Medicaid Expansion and Non-Expansion States

Characteristic	Medicaid expansion (n = 66,928)	Non-Medicaid expansion (n = 135,086)	Total (n = 202,014)
Age, y, mean (SD)	31.4 (7.7)	31.2 (7.6)	31.3 (7.6)
Sex, n (%)			
Male	46,512 (69.5)	94,279 (69.8)	140,791 (69.7)
Female	20,416 (30.5)	40,807 (30.2)	61,223 (30.3)
ZIP code-level median household income quartile, n (%)			
Quartile 1 (lowest)	19,113 (28.6)	47,583 (35.2)	66,696 (33.0)
Quartile 2	15,307 (22.9)	43,986 (32.6)	59,293 (29.4)
Quartile 3	16,776 (25.1)	29,850 (22.1)	46,626 (23.1)
Quartile 4 (highest)	15,732 (23.5)	13,667 (10.1)	29,399 (14.6)
Primary payer, n (%) (n = 365,837)			
Medicare	2,942 (4.4)	5,028 (3.7)	7,970 (4.0)
Medicaid	15,509 (23.3)	16,400 (12.2)	31,909 (15.8)
Private insurance	32,429 (48.6)	59,543 (44.2)	91,972 (45.7)
Self-pay	8,731 (13.1)	32,530 (24.2)	41,261 (20.5)
No charge	901 (1.4)	4,242 (3.1)	5,143 (2.6)
Other	6,176 (9.3)	16,939 (12.6)	23,115 (11.5)
Urban or rural residence, n (%)			
Large central metropolitan (>1 million population)	9,967 (14.9)	25,100 (18.6)	35,067 (17.4)
Large fringe metropolitan (>1 million population)	15,853 (23.7)	38,241 (28.3)	54,094 (26.8)
Medium metropolitan (250,000 to 999,999 population)	12,227 (18.3)	34,217 (25.3)	46,444 (23.0)
Small metropolitan (50,000 to 249,999 population)	8,127 (12.1)	12,937 (9.6)	21,064 (10.4)
Micropolitan	10,312 (15.4)	14,095 (10.4)	24,407 (12.1)
Noncore	10,442 (15.6)	10,496 (7.8)	20,938 (10.4)
No. of chronic conditions, n (%)			
0	16,329 (24.4)	35,530 (26.3)	51,859 (25.7)
1	16,784 (25.1)	34,949 (25.9)	51,733 (25.6)
2	12,615 (18.8)	24,782 (18.3)	37,397 (18.5)
3	8,174 (12.2)	16,256 (12.0)	24,430 (12.1)
> 3	13,026 (19.5)	23,569 (17.4)	36,595 (18.1)
Operative procedure, n (%)	37,537 (56.1)	79,405 (58.8)	116,942 (57.9)
Traumatic shock, n (%)	1,351 (2.0)	3,247 (2.4)	4,598 (2.3)
Injury Severity Score, n (%), (n = 367,039)			
Mild or moderate (0–15)	53,614 (80.1)	107,123 (79.3)	160,737 (79.6)
Severe (16–24)	9,470 (14.2)	19,931 (14.8)	29,401 (14.6)
Extremely severe (25–75)	3,839 (5.7)	8,024 (5.9)	11,863 (5.9)
Severe head or neck injury, n (%)	12,462 (18.6)	24,598 (18.2)	37,060 (18.3)
Injury mechanism, n (%)			
Cut or pierce	3,500 (5.2)	7,490 (5.5)	10,990 (5.4)
Fall	15,987 (23.9)	27,702 (20.5)	43,689 (21.6)
Firearm	2,150 (3.2)	5,193 (3.8)	7,343 (3.6)
Motor vehicle traffic	23,641 (35.3)	54,308 (40.2)	77,949 (38.6)
Struck by or against	5,208 (7.8)	9,769 (7.2)	14,977 (7.4)
Injury intent, n (%)			
Undetermined or unintentional	54,488 (81.4)	113,627 (84.1)	168,115 (83.2)
Self-harm	1,708 (2.6)	3,650 (2.7)	5,358 (2.7)
Assault	6,366 (9.5)	12,696 (9.4)	19,062 (9.4)

ARTICLE IN PRESS

eTable 3. Characteristics of Non-Hispanic Black Young Adult Trauma Patients in the Selected Medicaid Expansion and Non-Expansion States

Characteristic	Medicaid expansion (n = 32,502)	Non-Medicaid expansion (n = 66,457)	Total (n = 98,959)
Age, y, mean (SD)	29.5 (7.3)	29.9 (7.3)	29.8 (7.3)
Sex, n (%)			
Male	25,542 (78.6)	51,076 (76.9)	76,618 (77.4)
Female	6,960 (21.4)	15,381 (23.1)	22,341 (22.6)
ZIP code-level median household income quartile, n (%)			
Quartile 1 (lowest)	17,100 (52.6)	38,272 (57.6)	55,372 (56.0)
Quartile 2	4,911 (15.1)	17,783 (26.8)	22,694 (22.9)
Quartile 3	5,853 (18.0)	8,094 (12.2)	13,947 (14.1)
Quartile 4 (highest)	4,638 (14.3)	2,308 (3.5)	6,946 (7.0)
Primary payer, n (%) (n = 365,837)			
Medicare	1,163 (3.6)	2,386 (3.6)	3,549 (3.6)
Medicaid	13,522 (41.7)	11,118 (16.8)	24,640 (25.0)
Private insurance	8,725 (26.9)	17,259 (26.0)	25,984 (26.3)
Self-pay	6,436 (19.9)	23,218 (35.0)	29,654 (30.1)
No charge	280 (0.9)	2,915 (4.4)	3,195 (3.2)
Other	2,280 (7.0)	9,368 (14.1)	11,648 (11.8)
Urban or rural residence, n (%)			
Large central metropolitan (>1 million population)	17,914 (55.1)	23,765 (35.8)	41,679 (42.1)
Large fringe metropolitan (>1 million population)	7,515 (23.1)	19,512 (29.4)	27,027 (27.3)
Medium metropolitan (250,000−999,999 population)	3,426 (10.5)	11,767 (17.7)	15,193 (15.4)
Small metropolitan (50,000−249,999 population)	2,181 (6.7)	4,612 (6.9)	6,793 (6.9)
Micropolitan	960 (3.0)	4,173 (6.3)	5,133 (5.2)
Noncore	506 (1.6)	2,628 (4.0)	3,134 (3.2)
No. of chronic conditions, n (%)			
0	9,446 (29.1)	21,932 (33.0)	31,378 (31.7)
1	8,587 (26.4)	18,229 (27.4)	26,816 (27.1)
2	5,888 (18.1)	11,578 (17.4)	17,466 (17.6)
3	3,649 (11.2)	6,505 (9.8)	10,154 (10.3)
> 3	4,932 (15.2)	8,213 (12.4)	13,145 (13.3)
Operative procedure, n (%)	19,133 (58.9)	41,782 (62.9)	60,915 (61.6)
Traumatic shock, n (%)	1,182 (3.6)	2,406 (3.6)	3,588 (3.6)
Injury Severity Score, n (%) (n = 367,039)			
Mild or moderate (0−15)	26,514 (81.6)	54,397 (81.9)	80,911 (81.8)
Severe (16−24)	3,970 (12.2)	8,273 (12.4)	12,243 (12.4)
Extremely severe (25−75)	2,015 (6.2)	3,783 (5.7)	5,798 (5.9)
Severe head or neck injury, n (%)	4,556 (14.0)	9,055 (13.6)	13,611 (13.8)
Injury mechanism, n (%)			
Cut or pierce	3,742 (11.5)	5,655 (8.5)	9,397 (9.5)
Fall	3,949 (12.2)	7,829 (11.8)	11,778 (11.9)
Firearm	8,632 (26.6)	14,856 (22.4)	23,488 (23.7)
Motor vehicle traffic	7,849 (24.1)	21,390 (32.2)	29,239 (29.5)
Struck by or against	3,240 (10.0)	5,658 (8.5)	8,898 (9.0)
Injury intent, n (%)			
Undetermined or unintentional	17,108 (52.6)	43,394 (65.3)	60,502 (61.1)
Self-harm	393 (1.2)	925 (1.4)	1,318 (1.3)
Assault	13,255 (40.8)	19,753 (29.7)	33,008 (33.4)

eTable 4. Characteristics of Hispanic Young Adult Trauma Patients in the Selected Medicaid Expansion and Non-Expansion States

Characteristic	Medicaid expansion (n = 13,201)	Non-Medicaid expansion (n = 36,898)	Total (n = 50,099)
Age, y, mean (SD)	30.5 (7.4)	30.8 (7.5)	30.7 (7.5)
Sex, n (%)			
Male	10,658 (80.7)	29,186 (79.1)	39,844 (79.5)
Female	2,543 (19.3)	7,712 (20.9)	10,255 (20.5)
ZIP code-level median household income quartile, n (%)			
Quartile 1 (lowest)	3,740 (28.3)	16,602 (45.0)	20,342 (40.6)
Quartile 2	2,631 (19.9)	11,416 (30.9)	14,047 (28.0)
Quartile 3	3,925 (29.7)	6,695 (18.1)	10,620 (21.2)
Quartile 4 (highest)	2,905 (22.0)	2,185 (5.9)	5,090 (10.2)
Primary payer, n (%) (n = 365,837)			
Medicare	222 (1.7)	585 (1.6)	807 (1.6)
Medicaid	2,834 (21.7)	5,218 (14.2)	8,052 (16.1)
Private insurance	4,710 (36.1)	11,513 (31.3)	16,223 (32.5)
Self-pay	3,533 (27.1)	11,635 (31.6)	15,168 (30.4)
No charge	135 (1.0)	2,058 (5.6)	2,193 (4.4)
Other	1,596 (12.2)	5,830 (15.8)	7,426 (14.9)
Urban or rural residence, n (%)			
Large central metropolitan (>1 million population)	6,208 (47.0)	18,154 (49.2)	24,362 (48.6)
Large fringe metropolitan (>1 million population)	3,901 (29.6)	9,397 (25.5)	13,298 (26.5)
Medium metropolitan (250,000−999,999 population)	1,679 (12.7)	5,641 (15.3)	7,320 (14.6)
Small metropolitan (50,000−249,999 population)	472 (3.6)	1,369 (3.7)	1,841 (3.7)
Micropolitan	527 (4.0)	1,639 (4.4)	2,166 (4.3)
Noncore	414 (3.1)	698 (1.9)	1,112 (2.2)
No. of chronic conditions, n (%)			
0	4,694 (35.6)	15,405 (41.8)	20,099 (40.1)
1	3,682 (27.9)	10,221 (27.7)	13,903 (27.8)
2	2,136 (16.2)	5,384 (14.6)	7,520 (15.0)
3	1,212 (9.2)	2,799 (7.6)	4,011 (8.0)
> 3	1,477 (11.2)	3,089 (8.4)	4,566 (9.1)
Operative procedure, n (%)	7,123 (54.0)	22,478 (60.9)	29,601 (59.1)
Traumatic shock, n (%)	283 (2.1)	773 (2.1)	1,056 (2.1)
Injury severity score, n (%), (n = 367,039)			
Mild or moderate (0−15)	10,701 (81.1)	30,643 (83.1)	41,344 (82.5)
Severe (16−24)	1,843 (14.0)	4,573 (12.4)	6,416 (12.8)
Extremely severe (25−75)	656 (5.0)	1,678 (4.5)	2,334 (4.7)
Severe head or neck injury, n (%)	2,535 (19.2)	5,842 (15.8)	8,377 (16.7)
Injury mechanism, n (%)			
Cut or pierce	1,176 (8.9)	3,179 (8.6)	4,355 (8.7)
Fall	2,906 (22.0)	8,127 (22.0)	11,033 (22.0)
Firearm	1,064 (8.1)	1,825 (4.9)	2,889 (5.8)
Motor vehicle traffic	3,738 (28.3)	12,795 (34.7)	16,533 (33.0)
Struck by or against	1,369 (10.4)	3,268 (8.9)	4,637 (9.3)
Injury intent, n (%)			
Undetermined or unintentional	9,404 (71.2)	30,342 (82.2)	39,746 (79.3)
Self-harm	240 (1.8)	568 (1.5)	808 (1.6)
Assault	2,657 (20.1)	4,814 (13.0)	7,471 (14.9)

eTable 5. Characteristics of Young Adult Trauma Patients from ZIP Codes in the Lowest Income Quartile in the Selected Medicaid Expansion and Non-Expansion States

Characteristic	Medicaid expansion (n = 41,575)	Non-Medicaid expansion (n = 106,347)	Total (n = 147,922)
Age, y, mean (SD)	30.6 (7.5)	30.7 (7.5)	30.7 (7.5)
Sex, n (%)			
Male	31,140 (74.9)	79,173 (74.4)	110,313 (74.6)
Female	10,435 (25.1)	27,174 (25.6)	37,609 (25.4)
Race or ethnicity, n (%)			
Non-Hispanic White	19,113 (46.0)	47,583 (44.7)	66,696 (45.1)
Non-Hispanic Black	17,100 (41.1)	38,272 (36.0)	55,372 (37.4)
Hispanic (any race)	3,740 (9.0)	16,602 (15.6)	20,342 (13.8)
Other	1,622 (3.9)	3,890 (3.7)	5,512 (3.7)
Primary payer, n (%) (n = 365,837)			
Medicare	1,795 (4.4)	3,839 (3.6)	5,634 (3.8)
Medicaid	14,742 (35.7)	17,242 (16.3)	31,984 (21.7)
Private insurance	12,185 (29.5)	31,477 (29.7)	43,662 (29.6)
Self-pay	7,856 (19.0)	34,158 (32.2)	42,014 (28.5)
No charge	904 (2.2)	4,769 (4.5)	5,673 (3.9)
Other	3,781 (9.2)	14,575 (13.7)	18,356 (12.5)
Urban or rural residence, n (%)			
Large central metropolitan (>1 million population)	18,604 (44.7)	32,447 (30.5)	51,051 (34.5)
Large fringe metropolitan (>1 million population)	888 (2.1)	19,589 (18.4)	20,477 (13.8)
Medium metropolitan (250,000−999,999 population)	5,226 (12.6)	22,183 (20.9)	27,409 (18.5)
Small metropolitan (50,000−249,999 population)	3,994 (9.6)	8,396 (7.9)	12,390 (8.4)
Micropolitan	5,515 (13.3)	13,414 (12.6)	18,929 (12.8)
Noncore	7,348 (17.7)	10,318 (9.7)	17,666 (11.9)
No. of chronic conditions, n (%)			
0	11,425 (27.5)	31,820 (29.9)	43,245 (29.2)
1	10,980 (26.4)	28,359 (26.7)	39,339 (26.6)
2	7,622 (18.3)	19,009 (17.9)	26,631 (18.0)
3	4,789 (11.5)	11,522 (10.8)	16,311 (11.0)
More than 3	6,759 (16.3)	15,637 (14.7)	22,396 (15.1)
Operative procedure, n (%)	24,320 (58.5)	64,149 (60.3)	88,469 (59.8)
Traumatic shock, n (%)	1,208 (2.9)	3,112 (2.9)	4,320 (2.9)
Injury severity score, n (%) (n = 367,039)			
Mild or moderate (0−15)	33,500 (80.6)	85,938 (80.8)	119,438 (80.8)
Severe (16−24)	5,536 (13.3)	14,382 (13.5)	19,918 (13.5)
Extremely severe (25−75)	2,534 (6.1)	6,017 (5.7)	8,551 (5.8)
Severe head or neck injury, n (%)	6,606 (15.9)	16,947 (15.9)	23,553 (15.9)
Injury mechanism, n (%)			
Cut or pierce	3,701 (8.9)	8,328 (7.8)	12,029 (8.1)
Fall	6,819 (16.4)	17,883 (16.8)	24,702 (16.7)
Firearm	7,235 (17.4)	12,816 (12.1)	20,051 (13.6)
Motor vehicle traffic	11,593 (27.9)	36,869 (34.7)	48,462 (32.8)
Struck by or against	3,837 (9.2)	8,926 (8.4)	12,763 (8.6)
Injury intent, n (%)			
Undetermined or unintentional	26,769 (64.4)	79,292 (74.6)	106,061 (71.7)
Self-harm	765 (1.8)	2,109 (2.0)	2,874 (1.9)
Assault	11,453 (27.5)	21,123 (19.9)	32,576 (22.0)

eTable 6. Characteristics of Young Adult Trauma Patients from ZIP Codes in the Second-Lowest Income Quartile in the Selected Medicaid Expansion and Non-Expansion States

Characteristic	Medicaid expansion (n = 23,958)	Non-Medicaid expansion (n = 75,896)	Total (n = 99,854)
Age, y, mean (SD)	30.9 (7.6)	30.8 (7.5)	30.8 (7.5)
Sex, n (%)			
Male	17,620 (73.5)	55,332 (72.9)	72,952 (73.1)
Female	6,338 (26.5)	20,564 (27.1)	26,902 (26.9)
Race or ethnicity, n (%)			
Non-Hispanic White	15,307 (63.9)	43,986 (58.0)	59,293 (59.4)
Non-Hispanic Black	4,911 (20.5)	17,783 (23.4)	22,694 (22.7)
Hispanic (any race)	2,631 (11.0)	11,416 (15.0)	14,047 (14.1)
Other	1,109 (4.6)	2,711 (3.6)	3,820 (3.8)
Primary payer, n (%) (n = 365,837)			
Medicare	1,014 (4.3)	2,456 (3.2)	3,470 (3.5)
Medicaid	6,684 (28.0)	10,271 (13.6)	16,955 (17.0)
Private insurance	9,427 (39.6)	29,003 (38.3)	38,430 (38.6)
Self-pay	4,149 (17.4)	20,871 (27.6)	25,020 (25.1)
No charge	253 (1.1)	2,875 (3.8)	3,128 (3.1)
Other	2,306 (9.7)	10,227 (13.5)	12,533 (12.6)
Urban or rural residence, n (%)			
Large central metropolitan (>1 million population)	5,531 (23.1)	18,893 (24.9)	24,424 (24.5)
Large fringe metropolitan (>1 million population)	2,526 (10.5)	20,778 (27.4)	23,304 (23.3)
Medium metropolitan (250,000−999,999 population)	5,506 (23.0)	19,671 (25.9)	25,177 (25.2)
Small metropolitan (50,000−249,999 population)	3,434 (14.3)	7,073 (9.3)	10,507 (10.5)
Micropolitan	4,048 (16.9)	6,125 (8.1)	10,173 (10.2)
Noncore	2,913 (12.2)	3,356 (4.4)	6,269 (6.3)
No. of chronic conditions, n (%)			
0	6,416 (26.8)	23,260 (30.6)	29,676 (29.7)
1	6,267 (26.2)	20,334 (26.8)	26,601 (26.6)
2	4,424 (18.5)	13,092 (17.2)	17,516 (17.5)
3	2,723 (11.4)	8,171 (10.8)	10,894 (10.9)
> 3	4,128 (17.2)	11,039 (14.5)	15,167 (15.2)
Operative procedure, n (%)	13,722 (57.3)	45,822 (60.4)	59,544 (59.6)
Traumatic shock, n (%)	551 (2.3)	2,032 (2.7)	2,583 (2.6)
Injury Severity Score, n (%) (n = 367,039)			
Mild or moderate (0−15)	19,268 (80.4)	60,870 (80.2)	80,138 (80.3)
Severe (16−24)	3,301 (13.8)	10,644 (14.0)	13,945 (14.0)
Extremely severe (25−75)	1,387 (5.8)	4,379 (5.8)	5,766 (5.8)
Severe head or neck injury, n (%)	4,309 (18.0)	12,643 (16.7)	16,952 (17.0)
Injury mechanism, n (%)			
Cut or pierce	1,804 (7.5)	5,009 (6.6)	6,813 (6.8)
Fall	4,970 (20.7)	13,773 (18.1)	18,743 (18.8)
Firearm	2,157 (9.0)	6,099 (8.0)	8,256 (8.3)
Motor vehicle traffic	7,533 (31.4)	29,276 (38.6)	36,809 (36.9)
Struck by or against	2,017 (8.4)	5,809 (7.7)	7,826 (7.8)
Injury intent, n (%)			
Undetermined or unintentional	17,636 (73.6)	60,790 (80.1)	78,426 (78.5)
Self-harm	569 (2.4)	1,684 (2.2)	2,253 (2.3)
Assault	4,275 (17.8)	10,545 (13.9)	14,820 (14.8)

eTable 7. Characteristics of Young Adult Trauma Patients from ZIP Codes in the Second-Highest Income Quartile in the Selected Medicaid Expansion and Non-Expansion States

Characteristics	Medicaid expansion (n = 28,136)	Non-Medicaid expansion (n = 46,625)	Total (n = 74,761)
Age, y, mean (SD)	30.9 (7.6)	30.8 (7.6)	30.9 (7.6)
Sex, n (%)			
Male	20,606 (73.2)	33,587 (72.0)	54,193 (72.5)
Female	7,530 (26.8)	13,038 (28.0)	20,568 (27.5)
Race or ethnicity, n (%)			
Non-Hispanic White	16,776 (59.6)	29,850 (64.0)	46,626 (62.4)
Non-Hispanic Black	5,853 (20.8)	8,094 (17.4)	13,947 (18.7)
Hispanic (any race)	3,925 (14.0)	6,695 (14.4)	10,620 (14.2)
Other	1,582 (5.6)	1,986 (4.3)	3,568 (4.8)
Primary payer, n (%) (n = 365,837)			
Medicare	957 (3.4)	1,370 (2.9)	2,327 (3.1)
Medicaid	7,213 (25.7)	4,892 (10.5)	12,105 (16.2)
Private insurance	12,600 (44.9)	21,073 (45.3)	33,673 (45.2)
Self-pay	4,601 (16.4)	11,267 (24.2)	15,868 (21.3)
No charge	182 (0.6)	1,472 (3.2)	1,654 (2.2)
Other	2,501 (8.9)	6,406 (13.8)	8,907 (12.0)
Urban or rural residence, n (%)			
Large central metropolitan (>1 million population)	7,382 (26.2)	13,423 (28.8)	20,805 (27.8)
Large fringe metropolitan (>1 million population)	9,151 (32.5)	18,645 (40.0)	27,796 (37.2)
Medium metropolitan (250,000−999,999 population)	5,324 (18.9)	8,969 (19.2)	14,293 (19.1)
Small metropolitan (50,000−249,999 population)	2,859 (10.2)	3,727 (8.0)	6,586 (8.8)
Micropolitan	2,189 (7.8)	1,353 (2.9)	3,542 (4.7)
Noncore	1,231 (4.4)	508 (1.1)	1,739 (2.3)
No. of chronic conditions, n (%)			
0	7,758 (27.6)	14,885 (31.9)	22,643 (30.3)
1	7,283 (25.9)	12,422 (26.6)	19,705 (26.4)
2	5,106 (18.1)	7,947 (17.0)	13,053 (17.5)
3	3,188 (11.3)	4,791 (10.3)	7,979 (10.7)
> 3	4,801 (17.1)	6,580 (14.1)	11,381 (15.2)
Operative procedure, n (%)	15,680 (55.7)	28,247 (60.6)	43,927 (58.8)
Traumatic shock, n (%)	642 (2.3)	1,156 (2.5)	1,798 (2.4)
Injury Severity Score, n (%) (n = 367,039)			
Mild or moderate (0−15)	22,815 (81.1)	37,517 (80.5)	60,332 (80.7)
Severe (16−24)	3,809 (13.5)	6,413 (13.8)	10,222 (13.7)
Extremely severe (25−75)	1,508 (5.4)	2,692 (5.8)	4,200 (5.6)
Severe head or neck injury, n (%)	5,124 (18.2)	8,122 (17.4)	13,246 (17.7)
Injury mechanism, n (%)			
Cut or pierce	1,962 (7.0)	2,787 (6.0)	4,749 (6.4)
Fall	6,217 (22.1)	9,491 (20.4)	15,708 (21.0)
Firearm	1,969 (7.0)	2,779 (6.0)	4,748 (6.4)
Motor vehicle traffic	9,222 (32.8)	18,554 (39.8)	27,776 (37.2)
Struck by or against	2,405 (8.5)	3,366 (7.2)	5,771 (7.7)
Injury intent, n (%)			
Undetermined or unintentional	21,190 (75.3)	38,685 (83.0)	59,875 (80.1)
Self-harm	577 (2.1)	1,122 (2.4)	1,699 (2.3)
Assault	4,647 (16.5)	5,196 (11.1)	9,843 (13.2)

eTable 8. Characteristics of Young Adult Trauma Patients from ZIP Codes in the Highest Income Quartile in the Selected Medicaid Expansion and Non-Expansion States

Characteristic	Medicaid expansion (n = 25,421)	Non-Medicaid expansion (n = 19,108)	Total (n = 44,529)
Age, y, mean (SD)	30.7 (7.7)	30.7 (7.9)	30.7 (7.8)
Sex, n (%)			
Male	18,222 (71.7)	13,447 (70.4)	31,669 (71.1)
Female	7,199 (28.3)	5,661 (29.6)	12,860 (28.9)
Race or ethnicity, n (%)			
Non-Hispanic White	15,732 (61.9)	13,667 (71.5)	29,399 (66.0)
Non-Hispanic Black	4,638 (18.2)	2,308 (12.1)	6,946 (15.6)
Hispanic (any race)	2,905 (11.4)	2,185 (11.4)	5,090 (11.4)
Other	2,146 (8.4)	948 (5.0)	3,094 (6.9)
Primary payer, n (%) (n = 365,837)			
Medicare	684 (2.7)	547 (2.9)	1,231 (2.8)
Medicaid	4,781 (18.8)	1,436 (7.5)	6,217 (14.0)
Private insurance	14,547 (57.3)	10,577 (55.5)	25,124 (56.5)
Self-pay	3,246 (12.8)	3,776 (19.8)	7,022 (15.8)
No charge	59 (0.2)	335 (1.8)	394 (0.9)
Other	2,082 (8.2)	2,374 (12.5)	4,456 (10.0)
Urban or rural residence, n (%)			
Large central metropolitan (>1 million population)	5,399 (21.2)	4,814 (25.2)	10,213 (22.9)
Large fringe metropolitan (>1 million population)	16,908 (66.5)	11,150 (58.4)	28,058 (63.0)
Medium metropolitan (250,000−999,999 population)	1,741 (6.8)	2,555 (13.4)	4,296 (9.6)
Small metropolitan (50,000−249,999 population)	971 (3.8)	480 (2.5)	1,451 (3.3)
Micropolitan	312 (1.2)	92 (0.5)	404 (0.9)
Noncore	90 (0.4)	17 (0.1)	107 (0.2)
No. of chronic conditions, n (%)			
0	6,877 (27.1)	6,379 (33.4)	13,256 (29.8)
1	6,299 (24.8)	4,900 (25.6)	11,199 (25.1)
2	4,599 (18.1)	3,222 (16.9)	7,821 (17.6)
3	2,975 (11.7)	1,917 (10.0)	4,892 (11.0)
> 3	4,671 (18.4)	2,690 (14.1)	7,361 (16.5)
Operative procedure, n (%)	13,517 (53.2)	11,192 (58.6)	24,709 (55.5)
Traumatic shock, n (%)	573 (2.3)	433 (2.3)	1,006 (2.3)
Injury Severity Score, n (%) (n = 367,039)			
Mild or moderate (0−15)	20,431 (80.4)	15,370 (80.4)	35,801 (80.4)
Severe (16−24)	3,580 (14.1)	2,705 (14.2)	6,285 (14.1)
Extremely severe (25−75)	1,410 (5.5)	1,033 (5.4)	2,443 (5.5)
Severe head or neck injury, n (%)	4,851 (19.1)	3,554 (18.6)	8,405 (18.9)
Injury mechanism, n (%)			
Cut or pierce	1,472 (5.8)	872 (4.6)	2,344 (5.3)
Fall	6,198 (24.4)	4,376 (22.9)	10,574 (23.7)
Firearm	1,006 (4.0)	885 (4.6)	1,891 (4.2)
Motor vehicle traffic	8,908 (35.0)	7,484 (39.2)	16,392 (36.8)
Struck by or against	2,180 (8.6)	1,312 (6.9)	3,492 (7.8)
Injury intent, n (%)			
Undetermined or unintentional	19,950 (78.5)	16,232 (84.9)	36,182 (81.3)
Self-harm	577 (2.3)	470 (2.5)	1,047 (2.4)
Assault	3,211 (12.6)	1,678 (8.8)	4,889 (11.0)

ARTICLE IN PRESS

eTable 9. Risk-Adjusted Outcomes among Severely Injured Young Adult Trauma Patients in the Selected Medicaid Expansion and Non-Expansion States

	Expansion state		Non-expansion state			
Outcomes	2011–2013 (n = 9,495)	2014–2017 (n = 13,570)	2011–2013 (n = 18,846)	2014–2017 (n = 29,419)	Difference-in-differences estimate (95% CI)	p Value
In-hospital mortality	8.0	7.5	8.0	7.5*	0.06 (−0.72 to 0.85)	0.88
Failure to rescue	15.1	16.7*	14.8	16.2*	0.14 (−1.87 to 2.15)	0.89
Length of stay	9.9	9.2*	11.7	11.2*	−0.17 (−0.49 to 0.15)	0.29
Discharged to any rehabilitation	31.9	32.5	30.1	30.4	0.37 (−1.28 to 2.02)	0.66
Discharged to inpatient rehabilitation	19.0	21.4*	19.0	20.0*	1.44 (0.02 to 2.86)	0.04†
Discharged to a skilled nursing facility	3.3	3.4	3.3	3.0	0.55 (−0.10 to 1.21)	0.10
Discharged to home healthcare	9.6	7.8*	7.7	7.4	−1.62 (−2.65 to −0.59)	0.002†
30-d unplanned readmission	12.2	13.1	11.0	11.9	−0.003 (−1.90 to 1.89)	1.00
30-d return ED visit	16.9	17.5	18.6	18.8	0.41 (−1.85 to 2.66)	0.72
90-d unplanned readmission	15.6	16.4	14.4	15.2	−0.01 (−2.12 to 2.10)	0.99
90-d return ED visit	25.1	26.7	27.0	27.0	1.59 (−1.00 to 4.18)	0.23

Values are risk-adjusted marginal percentages.
*$p < 0.05$ vs years 2011–2013 in the same states.
†Statistically significant.
ED, emergency department.

eTable 10. Risk-Adjusted Outcomes among Severely Injured Young Adult Trauma Patients in the Selected Medicaid Expansion and Non-Expansion States: Results by Race or Ethnicity

	Expansion state		Non-expansion state		
**Outcomes by race or ethnicity**	**2011-2013 (n = 9,495)**	**2014-2017 (n = 13,570)**	**2011-2013 (n = 18,846)**	**2014-2017 (n = 29,419)**	**Difference-in-differences estimate (95% CI)**
In-hospital mortality					
White	6.8	7.1	7.4	7.1	0.67 (−0.32 to 1.67)
Black or African American	11.2	9.3	9.3	8.4	−1.07 (−2.64 to 0.50)*
Hispanic	6.4	5.2	7.5	7.0	−0.63 (−2.78 to 1.52)
Length of stay					
White	9.3	8.8	10.7	10.4	−0.11 (−0.50 to 0.28)
Black or African American	10.9	9.8	13.5	12.6	−0.22 (−0.92 to 0.49)
Hispanic	10.1	9.3	13.0	12.2	−0.02 (−1.01 to 0.98)
Discharged to any rehabilitation					
White	31.9	32.1	30.8	31.8	−0.78 (−2.88 to 1.31)
Black or African American	32.1	34.8	30.3	29.3	3.80 (0.10 to 7.51)*
Hispanic	32.8	31.2	24.9	26.5	−3.23 (−7.94 to 1.50)
Discharged to inpatient rehabilitation					
White	20.3	22.4	19.6	21.2	0.40 (−1.43 to 2.23)
Black or African American	17.5	20.4	18.5	18.6	2.89 (−0.24 to 6.01)
Hispanic	15.1	17.3	16.7	17.3	1.53 (−2.29 to 5.34)
Discharged to a skilled nursing facility					
White	3.2	3.4	3.3	3.1	0.39 (−0.43 to 1.21)
Black or African American	3.8	3.8	3.7	3.0	0.73 (−0.82 to 2.29)
Hispanic	3.1	2.9	2.4	2.1	0.14 (−1.67−1.94)
Discharged to home healthcare					
White	8.4	6.3	8.0	7.5	−1.68 (−2.91 to −0.46)
Black or African American	10.9	10.7	8.1	7.8	0.13 (−2.33 to 2.59)
Hispanic	14.5	10.1	5.9	7.2	−5.64 (−8.94 to −2.34)*
30-d unplanned readmission					
White	13.0	12.6	11.7	12.0	−0.63 (−3.27 to 2.02)
Black or African American	12.6	16.1	10.1	12.3	1.35 (−2.20 to 4.89)
Hispanic	8.5	9.0	9.7	11.1	−0.88 (−5.74 to 3.97)
30-d return ED visit					
White	17.4	16.9	18.3	19.2	−1.38 (−4.48 to 1.71)
Black or African American	17.4	19.8	20.5	20.5	2.38 (−1.89 to 6.66)
Hispanic	15.9	15.1	17.7	16.0	0.80 (−5.49 to 7.09)
90-d unplanned readmission					
White	16.3	15.8	15.0	15.3	−0.66 (−3.58 to 2.25)
Black or African American	16.1	19.4	14.4	16.0	1.64 (−2.34 to 5.61)
Hispanic	11.9	12.9	12.3	14.0	−0.68 (−6.23 to 4.87)
90-d return ED visit					
White	25.9	26.2	26.3	27.3	−0.66 (−4.21 to 2.90)
Black or African American	26.1	29.9	30.8	29.6	4.97 (0.06 to 9.88)
Hispanic	20.0	21.8	24.7	23.3	3.14 (−3.87 to 10.14)

Values are risk-adjusted marginal percentages. p Values shown are for difference-in-difference estimates within racial or ethnic subgroups.
*$p < 0.05$ vs difference-in-difference in non-Hispanic White patients.
ED, emergency department.

ARTICLE IN PRESS

eTable 11. Risk-Adjusted Outcomes among Severely Injured Young Adult Trauma Patients in the Selected Medicaid Expansion and Non-Expansion States: Results by ZIP Code-Level Median Household Income Quartile

	Expansion state		Non-expansion state		
**Outcomes by income quartile**	**2011–2013 (n = 9,495)**	**2014–2017 (n = 13,570)**	**2011–2013 (n = 18,846)**	**2014–2017 (n = 29,419)**	**Difference-in-differences estimate (95% CI)**
In-hospital mortality					
Quartile 1 (lowest)	8.2	8.5	8.4	7.9	0.86 (−0.44 to 2.17)
Quartile 2	8.5	7.2	8.1	7.3	−0.50 (−2.15 to 1.15)
Quartile 3	7.4	7.2	8.1	7.8	0.00 (−1.67 to 1.67)
Quartile 4 (highest)	7.2	5.7	6.7	6.2	−0.89 (−2.84 to 1.07)
Length of stay					
Quartile 1 (lowest)	10.4	9.3	12.1	11.6	−0.61 (−1.16 to −0.07)
Quartile 2	9.9	9.0	11.5	11.1	−0.51 (−1.17 to 0.14)
Quartile 3	9.6	9.2	11.6	10.6	0.54 (−0.14 to 1.21)
Quartile 4 (highest)	9.1	8.8	11.2	10.9	0.08 (−0.78 to 0.93)
Discharged to any rehabilitation					
Quartile 1 (lowest)	29.7	31.7	28.8	28.7	2.10 (−0.48 to 4.69)
Quartile 2	33.9	32.1	30.5	30.6	−1.95 (−5.29 to 1.39)
Quartile 3	32.4	34.4	30.8	32.0	0.72 (−2.90 to 4.34)
Quartile 4 (highest)	34.8	34.1	32.6	33.6	−1.74 (−6.52 to 3.05)
Discharged to inpatient rehabilitation					
Quartile 1 (lowest)	17.5	21.5	17.7	18.3	3.31 (1.11 to 5.51)*
Quartile 2	21.2	20.5	19.3	20.4	−1.83 (−4.71 to 1.05)
Quartile 3	19.4	22.2	20.4	21.1	2.10 (−1.03 to 5.23)
Quartile 4 (highest)	19.7	21.9	20.9	24.1	−0.97 (−5.12 to 3.18)
Discharged to a skilled nursing facility					
Quartile 1 (lowest)	2.9	2.8	3.4	3.0	0.25 (−0.74 to 1.25)
Quartile 2	3.4	3.7	3.5	2.8	1.01 (−0.35 to 2.37)
Quartile 3	3.6	3.8	3.2	3.2	0.17 (−1.31 to 1.65)
Quartile 4 (highest)	3.6	4.2	3.1	2.6	1.05 (−0.83 to 2.93)
Discharged to home healthcare					
Quartile 1 (lowest)	9.3	7.3	7.7	7.4	−1.63 (−3.23 to −0.03)
Quartile 2	9.4	7.9	7.7	7.4	−1.19 (−3.26 to 0.88)
Quartile 3	9.4	8.3	7.3	7.7	−1.55 (−3.78 to 0.67)
Quartile 4 (highest)	11.4	8.1	8.5	6.9	−1.83 (−4.82 to 1.16)
30-d unplanned readmission					
Quartile 1 (lowest)	10.7	11.2	9.8	11.7	−1.31 (−4.51 to 1.90)
Quartile 2	11.8	13.2	10.1	10.2	1.28 (−2.72 to 5.28)
Quartile 3	12.0	13.8	12.1	12.1	1.86 (−2.19 to 5.91)
Quartile 4 (highest)	16.4	16.7	13.9	15.3	−1.09 (−7.00 to 4.82)
30-d return ED visit					
Quartile 1 (lowest)	21.5	20.4	20.2	21.3	−2.12 (−6.33 to 2.09)
Quartile 2	15.8	17.2	19.8	19.2	2.10 (−2.48 to 6.68)
Quartile 3	15.1	15.3	17.6	16.8	1.00 (−3.53 to 5.53)
Quartile 4 (highest)	11.8	13.9	12.9	14.9	0.02 (−5.61 to 5.66)
90-d unplanned readmission					
Quartile 1 (lowest)	14.0	14.8	13.2	15.4	−1.45 (−5.04 to 2.14)
Quartile 2	14.7	16.9	14.6	13.6	3.17 (−1.25 to 7.59)
Quartile 3	15.6	17.0	14.7	15.5	0.65 (−3.81 to 5.11)
Quartile 4 (highest)	19.4	19.0	16.1	17.3	−1.62 (−7.88 to 4.65)

(Continued)

ARTICLE IN PRESS

eTable 11. Continued

Outcomes by income quartile	Expansion state		Non-expansion state		Difference-in-differences estimate (95% CI)
	2011–2013 (n = 9,495)	2014–2017 (n = 13,570)	2011–2013 (n = 18,846)	2014–2017 (n = 29,419)	
90-d return ED visit					
Quartile 1 (lowest)	29.4	29.9	29.2	30.7	−1.07 (−5.79 to 3.66)
Quartile 2	20.9	26.5	28.7	27.1	7.07 (1.89 to12.24)
Quartile 3	26.3	24.4	25.2	24.1	−0.75 (−6.12 to 4.62)
Quartile 4 (highest)	18.8	21.8	19.9	21.7	1.26 (−5.43 to 7.96)

Values are risk-adjusted marginal percentages. p Values shown are for difference-in-difference estimates within community income level subgroups.
*p < 0.05 vs difference-in-difference in highest income quartile
ED, emergency department.

eTable 12. Rate of Discharge to Rehabilitation among Young Adult Trauma Patients with Injuries Meeting Centers for Medicare and Medicaid Services Criteria for Inpatient Rehabilitation in the Selected Medicaid Expansion and Non-Expansion States

Discharge disposition	Expansion state		Non-expansion state		Difference-in-difference estimate (95% CI)	p Value
	2011–2013 (n = 56,196)	2014–2017 (n = 62,893)	2011–2013 (n = 104,344)	2014–2017 (n = 143,595)		
Discharged to any rehabilitation	27.9	30.6*	27.1	28.6*	1.13 (0.02 to 2.24)	0.04†
Discharged to inpatient rehabilitation	15.3	18.0*	15.0	16.7*	0.92 (0.01 to 1.83)	0.04†
Discharged to a skilled nursing facility	3.6	4.4*	3.6	3.3*	1.10 (0.56 to 1.63)	< 0.001†
Discharged to home healthcare	8.9	8.2*	8.4	8.7	−0.96 (−1.74 to −0.18)	0.02†

Values are risk-adjusted marginal percentages.
*p < 0.05 vs years 2011-2013 in the same states.
†Statistically significant.

eTable 13. Rate of Discharge to Rehabilitation among Young Adult Trauma Patients with Injuries Meeting Centers for Medicare and Medicaid Services Criteria for Inpatient Rehabilitation in the Selected Medicaid Expansion and Non-Expansion States: Results by ZIP Code-Level Median Household Income Quartile

	Expansion state		Non-expansion state		
**Discharge disposition by income quartile**	**2011–2013 (n = 56,196)**	**2014–2017 (n = 62,893)**	**2011–2013 (n = 104,344)**	**2014–2017 (n = 143,595)**	**Difference-in-differences estimate (95% CI)**
Discharged to any rehabilitation					
Quartile 1 (lowest)	26.1	28.8	26.2	27.4	1.52 (−0.41 to 3.45)
Quartile 2	29.3	30.6	28.1	28.9	0.49 (−1.98 to 2.96)
Quartile 3	28.5	32.5	27.0	29.6	1.29 (−1.30 to 3.88)
Quartile 4 (highest)	30.2	33.0	28.1	31.0	−0.03 (−3.38 to 3.31)
Discharged to inpatient rehabilitation					
Quartile 1 (lowest)	14.3	18.3	14.1	15.8	2.31 (0.73 to 3.89)*
Quartile 2	16.6	17.4	15.6	16.9	−0.57 (−2.61 to 1.46)
Quartile 3	15.3	18.6	15.3	17.2	1.46 (−0.67 to 3.59)
Quartile 4 (highest)	16.6	17.9	16.0	19.2	−1.88 (−4.65 to 0.89)
Discharged to a skilled nursing facility					
Quartile 1 (lowest)	3.0	3.2	3.8	3.4	0.63 (−0.17 to 1.43) *
Quartile 2	4.4	4.6	3.8	3.1	0.95 (−0.20 to 2.09) *
Quartile 3	3.9	5.0	3.1	3.6	0.69 (−0.48 to 1.85) *
Quartile 4 (highest)	3.5	5.7	3.3	2.6	2.79 (1.35 to 4.23)
Discharged to home healthcare					
Quartile 1 (lowest)	8.7	7.3	8.1	8.2	−1.51 (−2.75 to −0.27)
Quartile 2	8.3	8.5	8.7	9.0	−0.05 (−1.63 to 1.53)
Quartile 3	9.3	8.8	8.5	8.8	−0.84 (−2.53 to 0.84)
Quartile 4 (highest)	10.0	9.3	8.7	9.1	−1.09 (−3.28 to 1.10)

Values are risk-adjusted marginal percentages.
*$p < 0.05$ vs difference-in-difference in highest income quartile.

eTable 14. Risk-Adjusted Length of Stay Overall and by ZIP Code-Level Median Household Income Quartile in the Selected Medicaid Expansion States (Excluding Maryland) and Non-Expansion States

	Expansion state		Non-expansion state			
**LOS by income quartile**	**2011–2013 (n = 56,196)**	**2014–2017 (n = 62,894)**	**2011–2013 (n = 104,348)**	**2014–2017 (n = 143,628)**	**Difference-in-differences estimate (95% CI)**	**p Value**
LOS	5.05	5.12*	5.48	5.53*	0.02 (−0.06 to 0.09)	0.67
LOS by community income quartile						—
Quartile 1 (lowest)	5.25	5.21	5.62	5.67	−0.09 (−0.21 to 0.03)	—
Quartile 2	5.07	5.07	5.40	5.52	−0.11 (−0.26 to 0.04)	—
Quartile 3	4.93	5.14	5.38	5.36	0.23 (0.08 to 0.39)	—
Quartile 4 (highest)	4.57	4.80	5.27	5.36	0.15 (−0.05 to 0.36)	—

Values are risk-adjusted marginal mean number of days.
*$p < 0.05$ vs 2011–2013 in the same states.
LOS, length of stay.

eTable 15. Sensitivity Analysis Evaluating Risk-Adjusted Changes in In-Hospital Mortality among Black Young Adult Trauma Patients (1 State Excluded from Each Analysis)

	Expansion state		Non-expansion states			
**State**	**2011–2013**	**2014–2017**	**2011–2013**	**2014–2017**	**Difference-in-differences estimate (95% CI)**	**p Value**
AR	3.1	2.4	2.3	2.0	−0.50 (−0.89 to −0.11)	0.01
IA	3.1	2.4	2.3	2.0	−0.40 (−0.78 to −0.02)	0.04
IL	3.4	2.2	2.3	2.0	−0.90 (−1.37 to −0.43)	< 0.001
KY	3.1	2.4	2.3	2.0	−0.53 (−0.91 to −0.14)	0.007
MD	3.1	2.5	2.3	2.0	−0.32 (−0.70 to 0.06)	0.10
FL	3.3	2.5	2.2	2.0	−0.45 (−0.87 to −0.03)	0.04
GA	3.1	2.4	2.4	2.1	−0.42 (−0.82 to −0.01)	0.04
KS	3.1	2.4	2.3	2.0	−0.43 (−0.81 to −0.05)	0.03
MO	3.0	2.4	2.3	2.1	−0.44 (−0.82 to −0.06)	0.02
NC	3.1	2.4	2.3	2.0	−0.39 (−0.78 to −0.07)	0.05

Values are risk-adjusted marginal percentages.

ARTICLE IN PRESS

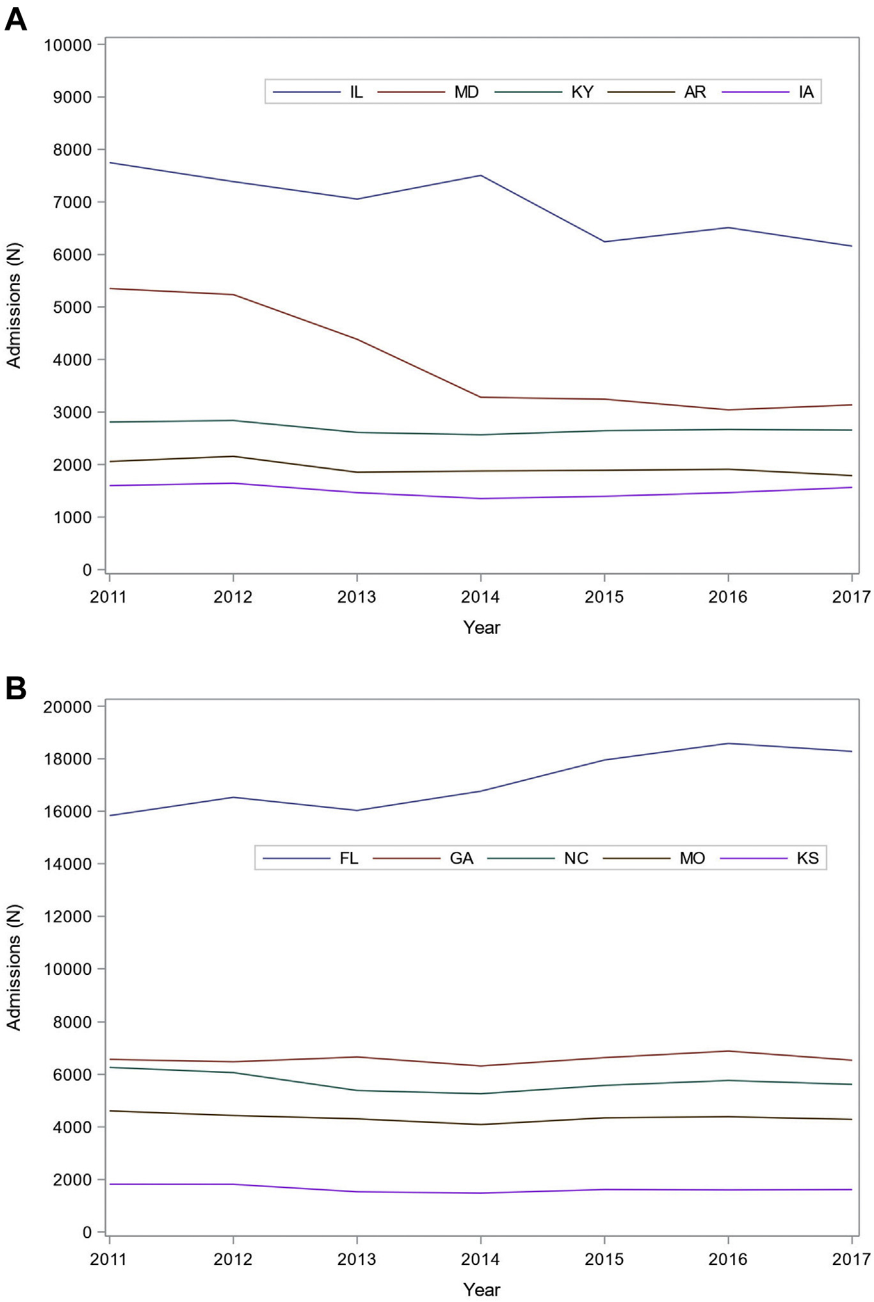

eFigure 1. Annual number of young adult trauma admissions in 2011-2017 in the selected (A) expansion and (B) non-expansion states.

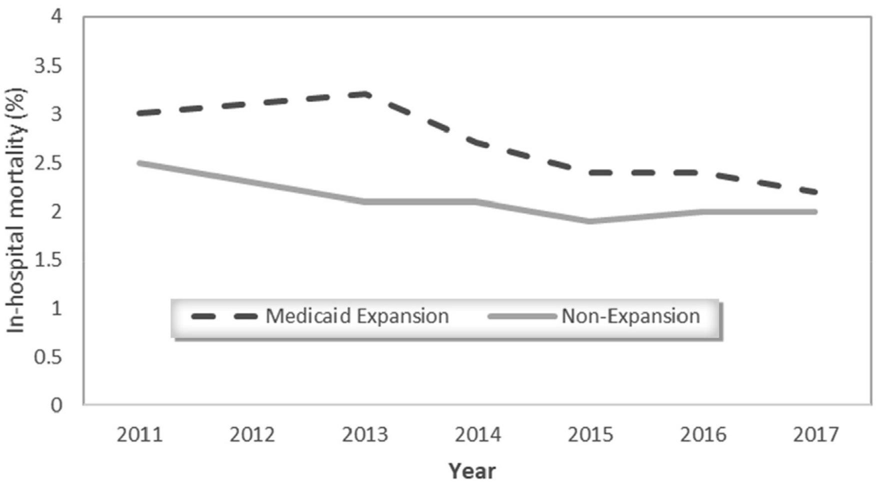

eFigure 2. Proportion of young Black adult trauma patients who died in the hospital over time, by state Medicaid expansion status.
